# Supplementary material for: Tuberculosis related barriers and facilitators among immigrants in Atlantic Canada: A qualitative study
Source: PLOS Glob Public Health. 2023 Jun 5;3(6):e0001997. doi: 10.1371/journal.pgph.0001997 (PMC10241363; doi:10.1371/journal.pgph.0001997)
Supplement: S1 Appendix — (PDF) [file pgph.0001997.s001.pdf]

S1 Appendix

Focus Group Discussions transcripts

Interviewers: Research Assistant 1 (**RA1)**, Research Assistant 2 (**RA2**)
1 **Researcher** in attendance

Focus Group Discussion #1 (In-Person)

Participants(origin): P.1.1(Africa), P.2.1(Africa), P.3.1(Asia), P.4.1(Africa), & P.5.1(Asia)

1. Tell me about your experience with tuberculosis in your home country.

P.5.1: "So my uncle had it, my father's brother...he is a general physician by himself...but about seven years ago, he found out that he has it...and we see him very bad...like in a bad condition...that when it comes active...it was very hard for him to breath when he lay down so he was sitting all the time...and his lungs filled with water...that when...like..a symptom of TB when we think about...and...uhm...they were take out the water...like he has to go to the hospital to take out the water from his lung out...and he lost a lot of weight.. Now he is fine...but he had treatment as the doctor said 5 or 6 months … and a lot of medication he has to take...yeah.."

RA1: "It is good that he is good now...so he knew that he had it when it became active.?"

P.5.1: "yes…because he is a doctor so when he feels the symptoms and wants to do a check...that is everything.. And if I doubt it is a TB...and I didn't ask so many questions at that time because it has been seven years ago so I didn't ask him many questions that time..but now I want to know ..like how it can spread and if we should be careful.. Or if we should get tested...yeah…so i want to know how much it is contagious...and if a family member has it..is it possible that there is like cancer or any other disease should family members watch out for it.."

RA1: "So I guess that was a question, right?"

P.5.1: "yes"

Researcher: "So I guess it is fair to address this here that there is active tuberculosis and inactive or latent tuberculosis and what you are referring to is active tuberculosis... because he actually showed signs and symptoms…what happens in those situations is...the people who came into contact with that person …need to be assessed."

P.5.1: "on which stage of the disease?"

Researcher: "No, I mean which country you are in..for example.. In many parts of Africa. Mainly they treat active TB...they just look out for signs and symptoms. Here in high income countries...like Canada...if somebody has active TB ...they are going to check everybody whom that person came into contact with and check the for possible exposure. If positive they are offered treatment to reduce chances of developing active TB later. In the case that you narrated..sometimes they keep you in a hospital for at least two

months… When they find out that you have active TB and they treat you with many different drugs...it is believed that after two months of treatment... the chances of transmitting the disease lessens..and then they continue administering medications to make sure that...you get rid of the bacteria from the body...and if the medication was effective then you are good to go..if the medication is not effective then they add some more medications..so treatment can go up to 2 years..."

P.5.1: "and could it spread or transmitted from..not disinfected..you know people back in our countries they are having tea or juices from the same bowl and it is not going into sanitizer...it is just getting rinse in the same bucket again and again…so could that be one of the reason?"

Researcher: "Predominantly by aerosols.. Coughing...so because it is airborne it is difficult for what's on a cup to enter our airways.. It will enter from the mouth..the problem when it's entered through the nose..so it is mainly things like coughing, laughing .. talking, sneezing.. that contribute to the transmission."

RA1: "Anyone would like to add something.."

P.4.1: "yeah..growing up I live next to the hospital..where they were taking care of patients with TB...so it was called TB annex.. That was the name of the hospital..so it is for patients with TB..that's from where I heard about TB when I was kid.. I don't know details about it…just was said..you are coughing like someone has TB.. like a joke..and then every time when someone goes to [Country name] refugee camp ...my sister started coughing and chewing blood.. And she went to the hospital and she was diagnosed with TB… so she stayed in the hospital for a couple of weeks..maybe months..I don't know.. But she was bad...and we used to visit her and bring her food.. And then after she came home and applied to come to Canada... They asked for a medical check and they found something in her chest X-ray...so the program took her a long time..usually it takes two years but it took for her 5 years… But she is finally here in Canada..."

Researcher: "are there any other contributions?"

P.1.1: "So I came from [Country name]..and personally I have never met in my circle or had a member of family who has TB…so I never have that experience with somebody.. So I never paid that attention..although in [Country name] it is very common..to have the infection..So until I spoke to you in the interview that we had..it sparked my interest..although I had not met or heard..but when you mentioned about the sleeping or latent TB..which I was totally unaware of...that there was something like that..my assumption was just TB is a viral infection that infect the person and you will get sick...but nothing about the latent..I could be an infected person...and not knowing about it...so I wanted to do some reading about it… and I was amazed that it is something ...definitely we should all get tested for.. Despite not having the signs ...because it is very easy to get... If we got in contact with someone who is active.. And.. you will be...easily infected by it…so...that really was an eye opener for me."

P.5.1: "So as he said a severe cough or people had…a...like it could be back home we heard about whooping cough or .some kind of cough ..people constantly coughing...but could it be related with TB or ..they should have some other symptoms with it...like blood...or?"

Researcher: "No, not always..and the symptoms are not always exactly the same.. Not everyone for example coughs up blood ..other people keep coughing and producing sputum..maybe that sputum has a different color... Then...they sweat at night…so it is not one thing...combination of things ...coughing ...sweating...and... ultimately, it has to be tested and detected...by the medical personnel..because if someone is coughing it doesn't always mean that person has TB...

P.5.1: "yeah and the other thing was…as we talked in the interview…so the BCG vaccine..I had it..all the family members had it..I checked that... And I was reading about it..it prevents us for fifteen years maximum... But we are older enough now and should we... I mean is it possible that we have latent TB in our system ...or it could be like...I mean we had the vaccine so we are prevented..?"

P.2.1: "excuse me..I would love to say something about that..your question with the history of TB.. TB was discovered in 1800s and that caused so many deaths of so many people...in 1940 medication was discovered to kill TB and during that time there was drastically reduced to (wasn't clear)...but later in the 1980s it was also discovered that the spread of TB was increased...which shows that sometime when we have cellular drugs for TB or whatever infection.. TB microbes or pathogens might grow resistance for that drug with time…so now back to your question.. Now after 15 years maybe we have to look for another vaccination for TB ...I remember a long time ago when we were very small …when I was in primary school it was mandatory to take TB vaccine...and we all took the vaccine.. And since then…till I'm still staying up... I haven't tested for TB though... But it is advisable for everyone even if we have been vaccinated against TB… we should go and check… go and recheck ... maybe do another test to see if we have latent because there are no signs...so my advice ...generally for everybody...is to go for check-up… We might be thinking that we don't have TB but it is somewhere hidden in the body so that would be my advice...for now"

P.4.1: "Is TB a tropical disease?"

Researcher: "not necessarily because you can find TB pretty much everywhere...but it is associated with poor living conditions like crowding because you can imagine if you live in a crowded area and there is somebody sick...it spreads like wildfire... It is everywhere so it may be more like in Sub-Saharan Africa, but... it is present in many parts of the world."

RA1: "and generally speaking in any country for medical staff because they are more exposed to patients ...I remember I had needle stick injuries while I was treating my patients and they did the TB test for me …so any medical staff at any part of the world they are asked to do the test every two years or sometimes every year..it depends.. I think it is different from one country to another… but all the medical staff do the test regularly… In the middle east...I only heard about one of my relatives...he is a nurse and he got TB.. and actually, he had active TB long time after he retired..."

P.5.1: "and is there an age group..like do we find it more in younger children or in older kids ...or adults..?"

Researcher: It is across the life span.

P.1.1: “is there a possibility to get it for a second time or it’s one time ..?”

Researcher: “yes, it is possible...there is no lifetime immunity for TB...”

Researcher asking **P.3.1** about any experience with TB back home.

P.3.1: “no not actually in Asia I didn’t hear about TB and growing there I took very strong vaccinations.. And I don’t remember anyone had TB from my circle...but of course they are more vulnerable there for the reasons we talked about.. Like living in close proximity and so on.. Yeah..so my personal experience with active TB is good fortunately...”

Researcher: “and that is good news...hhh..”

P.3.1: “hhh yes… So the vaccination system became really strong in the last 25 years …”

P.2.1: “can we say someone with good immunity can actually survive with TB spread? ...can we say that?”

Researcher: “Sure.. if your immune system is strong and able to.. If you are infected..it is able to contain it.. sometimes or in those situations you end up with latent TB...because your immune system is able to contain it… But when your immune system goes down… Then the sleeping TB may start growing and cause problems...”

RA1: “and sometimes you may get another infection and it became opportunistic...”

Researcher: “Yeah you might get another disease, which compromises your immune system...then...so when it lowers your system then ...you are now at higher risk of developing active TB from latent TB”

<u>2. Why would some people not want to get screened for TB?</u>

P.3.1: “I think that’s a question like again nobody wants to..proactively… Like all human beings..need to be pushed to get tested...when it is mandated… from my personal experience with TB I can say..I got diagnosed with Leukemia this year..so of course my immune system..was really really...so Dr.[physician name] was seeing me for the last 6 months..so I was diagnosed with latent TB ...I never had symptoms.. It was residing in my body..but I had to go for multiple tests because of my condition…and I found out that I have it..but if you really ask me if I really want to be tested for TB..unless the symptoms came up…I don’t think that I would be tested for TB alone...unless I have to...but for TB as TB alone..no...it is just accidently I got tested ... and I took the treatment for four months and now I am fine...”

RA1: “but why do you think so? I mean why do you think people don’t want to be tested?”

P.3.1: “I think..it is just my personal thinking.. People generally don’t seem to have the symptoms in general...like coughing or sneezing … it could be a very seasonal thing so I would take Tylenol or Advil or whatever and I am done… And it goes away… And

generally if your immune system is good enough…as we said before...generally speaking...it might kill your coughing... sneezing and life is back to normal...unless your coughs or sneeze last for a longer time...so the symptoms are so subtle unfortunately for TB.. unless you are coughing blood or something like that..I think people might take it lightly… I assume not many people had that..but the other symptoms include fatigue.. coughing or sneezing. People take it lightly ...unless somebody or a medical professional tells me you need to get tested.. I try to avoid it..”

P.2.1: “Just like what you mentioned, P.1.1 is... The nonchalant attitude from people...people will be like it doesn't really matter.. And even if people want to get tested...sometimes..what the society will say.. You know.. It pouts at you for getting TB.. Are you going to have a supporting family member who will support you genuinely? Or your friends are going to stay with you or they will run away because you have TB...and they know even a small spread from you…can make them infected...so society ...uhm...is a factor…you know and everything coming to the mental health of the individual who is infected... The family running away..you know..nobody is coming to help..nobody is ready to..to..to..share your pain...you know emotionally ..so everything comes back.. To the person who is infected because… You will be stressed out.. You know.. Mentally.. such an individual will stress out..even if this person is taking drugs..you know… It might not be effective because ..you are not..getting the kind of support.. From a family member.. Or you're stigmatized. It's traumatic... I think that is one of the reasons...”

P.4.1: “I just want to add that..for me it depends whether I am in Canada or I'm in [Country name]... I think I would do the test in Canada because the health is good.. Financially I will be ok… Since I came to Canada, I do a medical check-up severy year. All my life in [Country name] I never looked for trouble in myself..hhh...or go to the hospital looking for sickness...because you have to pay...for the tests..and even if you are diagnosed you have to pay for the treatment… So why do you...and it is not just for TB.. for any kind of sickness…and you don't have the money... And the process...even in Canada..and I have to wait 2 hours somewhere to do the TB test.. I will not do it…like I want the smooth process...because for example… I don't like...hospitals..if the process is difficult for me ..for example when I see COVID test...and...putting things in nose...I will not do COVID test.. Like just..just.. Because of the process… maybe if I will travel and I have to do it then I will do it..but if they say there is COVID test and it is free come to do it..I won't do it..just because of that...if COVID test was like I spit somewhere and get the result ..I will do it..so it is more individual kind of thing.. And how the test could be..the cost of the test..and the treatment if I am diagnosed.. what is the cost of the treatment..can I afford it… What is my own mental status... What If I am diagnosed positive? How I will handle it..how can I find the help..all the possible things.. And what i am saying is not just for TB..it is the same for HIV... or other kinds of diseases... That's why people might not take the test...”

RA1: “of course...we cannot neglect the financial factor...if someone knows that he will pay for any medical check...he will say I am fine..my family are now in [City name] and they don't have medical insurance...everytime when something happens and I say you have to go check..they say no we are fine..hhh..”

P.1.1: “I agree with that completely..like… Back home you don't visit the doctor unless you cannot move..or clearly sick.. Then you go and get treatment..otherwise..you just take tylenol or ..anything..and you will be fine…but here any little headache..it is readily..like

the service is free of course...I mean the financial factor..and also the kind of service available..so you are encouraged to go and get checked out... To be on the safe side… My other point would be.. That you would not be checked because you don't know the severity of certain diseases...like in my case... I would never go get tested or educate myself about TB because I've never experienced or got personally in contact with somebody who actually had the disease..it is after speaking to you... I knew that I could be having sleeping latent TB ...that made me think that I should also go get the test...so I was never informed about this thing..."

Researcher: "I was just going to add that...we are fortunate in Canada in the sense that we don't have to pay for the test or the treatment. So when one is diagnosed with TB ..the treatment.. the healthcare system is going to take care of the treatments, which is not always the case in low-income countries..."

P.4.1: "One thing..even back home.. There is a lot of ...like..foreign funding for ..TB or malaria.. And those kinds of things..so the treatment will be subsidized.. But still..you have time away from work...time away family..there will be a lot of other things to think about.."

Researcher: "You brought up a good point..most TB programmes receive international funding to help with treating TB...and this lowers the cost...."

P.3.1: "and everyone looks at things..like..I am talking from my experience.. As I said..I was diagnosed with latent TB as a side thing in addition to my main treatment.. The same situation ...in [Country name] that is where I came from.. I have to stress about what drs say because I might think they are saying this to take more money… Because the basic trust in the healthcare system of our original countries is missing... And that is one of the reasons that we don't want to go get tested… I would check my bank account before I go to the hospital and get myself treated.. Because most of the time we cannot afford it honestly.. A close friend was telling me...if I was diagnosed with Leukemia in [Country name]...I might not get treated.. Maybe I will say....let me just live the number of years I live... As opposed to getting treated there.. Because they will knock me off honestly .. so...yeah…geographically where you are located makes a huge difference."

P.4.1: " not directly related to the discussion...but relatively...In [Country name] when people are sick.. [Country name] is one of the places they go to get treated...hhh..."

P.3.1: " relatively...hhh"

P.4.1: "hhh..yeah when compared to America or Europe...like if you cannot afford going to America or Europe then they say there is a hospital in [Country name]...you go to..."

RA2: "So one thing in (somewhere, I think Canada), one of the main points, the trust and confidence in the system.. But from what I was hearing during the interviews. Many people were not aware of latent TB. (recorder is not clear here) so some of them were grateful because they didn't know about it…"

RA1: "I did a TB test a couple of weeks ago because I was applying to a university…so my husband was like...aha so I might have it.. Why don't I go with you to check

myself...because he didn't know about it… So yeah you are right…most people aren't aware of it..."

P.5.1: "just like I said...it was my uncle who had it and we spent a long time close to him… when he was sick..so we should all get checked ... so all my family members should know that we should check because we all might have latent TB..."

P.5.1: "so yeah if we have it and it needs to be treated at this stage.. It will take less time of treatment and…less money… hhh…"

3. Why would some people who have TB not want to tell their friends or neighbours?

P.4.1: "because of stigma"

P.5.1: "she said it...because of society.. You know...everybody gonna run away.. And don't wanna sit next to us... That could be the main reason..."

P.3.1: "I think it comes to the knowledge part..like..firstly a lot of people don't even know the difference between latent and active.. Including me..when I was diagnosed.. I was like...OO TB... is that?... Doctor said nothing...nothing to worry about...it just latent TB.. Every one out of 4 people has it...but they don't get tested… so there is a complete lack of knowledge about it… about the difference..and how we talk about TB…and how deadly it is...maybe it is...if it's active and in an advanced stage.. And the fear of isolation from your close ones… And you don't want to end up like that… When I had latent TB I didn't tell too many people about it honestly...because I didn't want to think it is infectious like active TB...because people don't really know the difference..."

P.4.1: "I just remembered something now...I think two years ago.. Visiting some family members in [City name].. One of the ladies she went to apply for working at a nursing home.. And she wanted to do the test.. And I think ...I don't know...at that time...they said..she has something..with TB.. and there was a huge argument ..that we all have TB … because we are from that region...we all have it in our blood..I remember that message coming back now..because..she is from my country and for the job she has to do the test... And there was something related to TB ... and we were discussing...for me once you have coughs with blood then you have TB...hhh…"

P.5.1: "and that reminds me..I am working in childcare and I moved from Ontario to here… and working in Ontario for three years ...we get tested... so I got tested twice...yeah…yeah...but just from the blood test..no X-ray..."

Researcher: "and the blood test is more sensitive. So, the x-ray will pick up active TB...the blood test will pick up latent TB…to predict how likely you will progress to active TB"

P.5.1: "so it is mandatory for our color...for people from outside Canada if you are working in childcare…to get tested..."

Researcher: "so you are tested...hhh..."

P.5.1: "yeah I just remembered it now...hhh...here in New Brunswick they didn't ask me

to get tested..."

RA1: "I remember one of the interviews that I did, someone asked if they start doing the test for people who want to work in the food industry..."

4. What would make someone with latent TB infection want to take treatment?

P.1.1: "the financial factors. Like if the person could afford it and it is available...they'll definitely go for it...if it is not..they cannot afford it..they will avoid taking it..."

Researcher: "How about we consider the situation in Canada like here in New Brunswick…so the cost of fees...hhh"

P.4.1: "so for example…like participant 3 situation...if I had one health issue..and it came up... and the dr says...or he gives the prescription or the treatment…I will definitely get the treatment...or once I do the test…for example you are doing it and I volunteered .. And I am advised to get the treatment...you can do it..and you prescribe it to me and my insurance covers it...I will for sure take it… otherwise I don't have a reason to not take it..."

Researcher: "what would be the motivation to get treated?"

P.3.1: "so it won't become active TB...at later years"

P.2.1: "and another thing is latent TB ...if not treated..it is death related... And no one wants to die…so you get treated."

Researcher asked if anyone had something to say.

P.3.1: "what we are lacking again..it is the knowledge piece..if somebody is properly educated...let's say as part of the immigration system.. Like when you enter a new country…you've been told...that you need to get treated.. Like if in Canada or New Brunswick they told you there is something you should do, because you have the advantage of getting that for no cost...and also how much time does it take.. And how intrusive or non-intrusive it is...if it's just taking a pill for a couple of months...with no cost..or minimal cost..I don't think there should be a problem."

P.4.1: "just a question...I don't know if you put that out..so during immigration you do a medical exam...like I was here in Canada I did a medical check… And it was late so I have to do another medical check-up...I don't know if they look for latent TB in the medical exam."

Researcher: "No they do not..because the only tool they use to screen for TB for immigration purposes is X-ray…and the chest x-ray is looking for active TB...so.."

- What would make someone with TB Not want to take treatment?

P.4.1: "I think in Canada people will take it… (everyone confirms)..but back home..again ...like ...I remember a case of my sister in law.. I noticed that she knew that she had it...like before...but she tried to keep it as a secret. Until she was really sick and everybody

knew..so that's one of the things.. people might have it..or they have trouble cough and everything and know that they don't feel well..but they keep it private or tell somebody close to them ...and it is a cultural thing..like people don't walk around and meet people and say Oh I have TB or my father has TB..No..it is not like that...it is like a family secret.. Like even in the family you only tell older people..it is like a secret…for instance my sister in law..when she got TB and went to the hospital.. we didn't tell her kids all the details...we just said sorry your mom is sick, and you cannot see her… Only this person can go and see her...but we didn't tell the details..so it goes all around the neighbourhood."
Somebody asked "Why?"

P.4.1: "I don't know the reason..it is a cultural thing..I don't why we do it but you don't tell people..like..like..I know somebody in Canada who told me about a relative who had HIV…but from where I am...you will never know… Like only a few people in the family know.. And like I said It is a cultural thing... And a main thing in our culture ...I don't know why we do it… But it is like that…"

P.3.1: "it is the social stigma..."

P.4.1: "yes…because even if you are cured from TB..people still describe you like that one of TB ...hhh...people even tell their children ...don't eat at that house they have TB...in their house..like if you have business…or (recorder here wasn't clear)..or something...so we just keep it.."

P.2.1: "So I think that is a major reason why in Africa generally ...we don't really get problems solved... When I came to Canada people are kind of open minded… people will tell you I am diabetic...I am hypertensive.. In Africa..where I came from..[Country name]..where I came from..if you are hypertensive... It is the family secret... If you are diabetic...it is...you know...you have to keep in the family... We are kind of secretive... We love to protect the image of the family... And not that...we don't feel bad about it…we feel so honoured... Like someone has a problem and we have to like.. Support that person.. And protect that person's image. But in the long run...it will come to affect all together as a family… but it will only be such a good...to...to help the individual to get access to health care.. You know..when the person is ready to go to hospital...are we ready to support the person through all this..and get the problem solved.. But I noticed here in Canada..people…like.. are open minded... You are diabetic..fine...are getting the treatment...yes..but I think it is becoming .. people are letting out so many things right now..so I've seen changes even within families.. When you have issues...speak out...people aren't ready to talk..because of what the society will say.. Visible stigmatization... You know people have been stigmatized for having particular health disorders.. So I think.. the solution..is the person..to speak out… Are you infected..yes..talk about it...to people who can protect you..but not to everybody..."

RA2 said something but her voice was very very low. But it was about because of the lack of knowledge they stigmatized families...even if they are cured.

P.2.1: "yes... even if you are cured..be careful who you tell..to who you talk to."

P.4.1: "and if you are getting married..and they know…hhh..."

P.2.1: “HEY...hhhhh…you don’t mention it...you know.”

RA1: “so I know someone and she was diagnosed with latent TB..but she was like... the treatment is lengthy… they told her that it might go up to 6 or 9 months to be treated.. And she was trying to talk to the Doctors because she cannot take antibiotics for that long.. So this one of the cases I know...and they tried to convince her...”

Researcher: “so the treatment for latent TB like I said... It used to be 8/9 months...now they are using drugs, which can lower it to 6 or even 4 months… So this drug was there before...previously they used to treat it with isoniazid... Isoniazid you take it once daily for 9 months... Now they figured out they can actually use other drugs like rifampicin for a shorter period, which helps with compliance.

<u>5. Is there anything else that you would like to share about latent TB infection?</u>

P.3.1: “one thing which the medical facility can do..when someone is diagnosed with latent TB...to educate the person a little bit more...it terms of... What latent TB is?..it is something that is very common...that supposed to be there..from my personal experience... The Doctor did tell me it is what it is...but a bit more information would have helped in terms of...keeping you calm and not really getting locked up..because of that...because I ended up doing a lot of google search when I was in the hospital.. And found okey that is it… nothing to worry about...but a little more the medical professional also can do when somebody is diagnosed with latent TB ...that would be really helpful...yeah...that was not my primary treatment that is going on...it was side treatment...but a little bit more information would’ve really helped...”

P.2.1: “something just came to my mind right now, I think…one of the things..or.. One of the factors..that actually.. Helps someone with latent TB ...is… Good diet…when someone..is diagnosed with latent TB.. other thing that taking the drugs..are you eating right?... You know...even if you have been told to use drugs. I believe there are things that you can add to your diet..that can redeem the spread of the infection..or can actually let the infection powerless... Like introduction of herbs into food...like garlic..ginger..to what we eat.. Sometimes it can help to redeem the effectiveness. Of the microbes on the body...”

RA1: “they also recommend taking yogurt or probiotics to replace the good bacteria that is killed by the antibiotics.”

Researcher: “so I think your point about the medical facilities providing more information about TB when detected is well taken.. Do you think there is a role..of the health care professionals in general… to provide some awareness..even before.. Someone tests positive?”

P.3.1: “absolutely yeah.. It depends at which form..or stage you want to do that...like as an immigrant… Or whatever ..I am sure there is a way to do that.. Like through what the newcomers center does something for the newcomers...or whatever way to do it more proactively. Not just when it is detected..and when it is detected..I am sure it is smart to give a little bit more information about what it is ...it would help..”

P.2.1: "I don't know if that is what he meant but advocacy is very important. And being in this kind of environment ...having different people from different countries..cultural beliefs and languages.. If it would help this research..I would suggest that if we can..you can include.. The use of different languages.. To advocate about TB...like I am from [Country name]..and from part that's good at [language name]...my language...so if you need someone.. To tell people from my own community in Canada.. About TB...in [language name]. I would volunteer and I know a lot would do it for the community."

P.5.1: "like sitting here and we talk and know about it.. I want to go back to my country and gather the people and educate them.. What it can be and how you should treat it…or what you should do.."

That concludes our discussion, thank you for your time.

Focus Group Discussion #2 (Virtual)

Participants: **P.1.2, P.3.2** & **P.4.2**(Africa), **P.2.2**(South America), & **P.5.2**(Asia).

RA1: Can you tell us about your experience with tuberculosis in your home country?

P.2.2: So, I am from [Country name], this illness was like eradicated because we were vaccinated since we were born. So far, use to be eradicated, like exterminated, no one has it, but now is different, the illness is start to coming again maybe because we don't have vaccination. I don't know if you hear about the crisis that we are having at this moment in [Country name]? There is no vaccination and people are starting to get it again.
So, my experience about this illness was with my dad. He was living in one province, in the north of [Country name], and he never got the vaccine because he was born in 1939/8, he is 80/81 years old now. But, at that moment we didn't have the vaccine. Anyway, the thing is that he had a neighbour, at that moment, when he was 6 years old, and this kid had the illness. They didn't know that. He was playing with this child; they were very good friends. Both kids were 6 years old, my dad and his friend. After one year the kid dies, her mom dies too, and they get to know that they had this sickness, tuberculosis. After 10 years, my dad went to [Name of city], which is the capital of the country, to start high school there, because he wanted to be an engineer. So, it's like a technical school and you need to be tested for anything. He got tested for this illness, and he had a reaction on his skin, on the part that he got the test. He told me they put like water on his arm, close to the elbow, and when the water touched him, the reaction was like a very big circle, like a coin, but very big, and everybody was saying "He has tuberculosis." The thing was he got an X-ray and he was clean, and he never suffer, he never had anything. So, he thinks that maybe he can have the latent TB because he never got the symptoms. I mean, he is alive, he is very healthy, and he never gets sick for anything. He is very healthy.

Researcher: Thank you for that contribution. Any other contributions from…

P.4.2: Hello everyone. So, what I know from my country, [Country name], that's where I came from. What I was saying is that, none from me, apart from many miles ..or from the media, once in a while, I didn't hear much of it. They talked about that tuberculosis is high in the country, maybe in the 2000 late '90. Personally, I don't know anybody with that disease, I don't really know much. But, by the time I started to have kids and going to hospitals, I realized that infants, when babies are born, we were asked to give the BCG vaccine to the babies. So, when I started going to the hospitals, that is when I started seen adults, you know? Even when I see them, I didn't know that they had tuberculosis, because the look of those sick people, they are in a different place, because it's a big hospital, it's a general hospital. You see them, they talk about it and because I went often to those hospitals, for one thing or the other, you take your baby for different vaccines. I begin to see different people that have tuberculosis, and I asked questions, why you see them, it's already shown over them. I begin to ask questions. If this is tuberculosis, if we are getting BCG vaccine for the babies, how can this people had, why did they get here? From there, I see that those people with the mental

problem, and the country was that people, jinx, you know, they don't regret. When they attack, because of the common, seems to be common symptoms, like cough, like sweeting, because normally they sweet, people sweet, because it's a hot region, those things become common. So, when they attack with those disease, like TB that it's in question here, they think is one of the common things, and they begin to treat it at home, or they go to common pharmacy, you know like nearby pharmacy and they get drugs to treat cough, or to treat maybe whatever, common symptoms they are feeling, or sometimes they go to a traditional healer. You know, I am talking about my country, where I come from, right? So, they go to places that…, So, when they go to treat those things without coming out, without clearing it, without going to the right place, that it's one of the problems. They don't go to the right places, the hospitals, they were not vaccinated, they think it's one of those common illness and they are treating it wrongly, they don't speak out, they treated at home, they go to wrong places to treat it, at the end of the day, when the elements are really into the system that is when they go into the hospital. So, the things of late diagnoses, or late treatments and it's all about them, they lose weight….this is how somebody suffering tuberculosis looks. This is how they look, so the look was scary, so slim, so, looking really sick. So, I will say that from where I come from, we hear that is common, but I really get to know people that has the disease on later on, and that was when I was able to get my children vaccinated. So few people that I see, and I asked questions, and those were some of the answers I got. And lastly, when I started go to the hospital, because before having kids, I wasn't really going to the hospital, I used to see posters that was how I got to know that the news was real, that tuberculosis was higher. Because anywhere you go in the General Hospital you will see posters, they would draw, the way they illustrated, you do it for those you have anything close to that disease, they illustrated, they will give the common symptoms, they will put it on the posters, so I think that help along the line where mothers make sure to get their children vaccinated. So, that it's what I submit for now.

Researcher: Thank you so much for that…mmm, are there any other contributions on this particular subject?

P.1.2: So, my experience with tuberculosis, I would say personally, I can't really say I know someone one on one that had the illness. I knew growing back in the '80 that were a lot of commercials on TV, back in Legal [Country name], telling people about tuberculosis, this is real, get treatment on law, like one on correspondence rightly mention, when kids are giving back to in [Country name]. I see regimen of vaccination that they have to take, and tuberculosis vaccine it's one of them. So, mid '80, late '80's you see a lot of commercials, when you see someone coughing beside you, you said, "Oh! Maybe you have tuberculosis." Personally, you hear it, but for you to say, "Uh! I know someone that has tuberculosis," it's difficult to say. Especially if you are not part of [Country name], where Hammer time weather or dry air drips weather it's very common, people tent to cough a lot. So, coughing and having an assistant dry cough wasn't common please. So, once you cough one or twice, maybe you have tuberculosis, but nobody really…I wouldn't say nobody took it seriously, but a lot of information going around the country, people actually took it more serious when they ended on the hospital. I would say, from my own personally experience, I see the

government putting a lot of ads, putting a lot of posters in the hospitals, I mean, telling pregnant woman to watch out and take care of their selves, wash their hands, usual keeping hygiene and things like that. But if you ask me what causes tuberculosis? What are the symptoms? I'm not able to tell you. What I knew for the fact that the information was there back then in West Africa Region [Country name]. How people are being treated? I would say yes, because once you go into any government hospitals you see posters talking about HIV/AIDS, talking about tuberculosis, talking about hepatitis, and I knew people do get treated for that. But personally, how many people do you know? for the fact I knew almost everybody who had malaria, for tuberculosis I can't really say. So, that is my experience with the disease. Thank you.

Researcher: Thank you for that contribution. So, what I hear is because some commonalities in the symptoms, people either discount or ignore the symptoms or go for to sick medication for things like a simple cough, not knowing they may have something more serious.

All right. I think maybe we can move on, **RA1**.
RA1: My second question. Why would some people not want to be screened for tuberculosis? Like if we ask someone, some people they won't volunteering go for the test. They say, if I had to be tested I would do it, but I don't like to go for the test. Why do you think so?

P.1.2: Why would someone won't go for the screening in Sub-Saharan Africa or Canada?
Researcher: In general.

P.1.1: So, in general, from my personal experience, I would say any type of sickness, they say if it is wild spread disease or sickness, people feels stigmatized. Right, I remember back in '85 when the HVI/AIDS virus broke out, nobody wanted to hear that you were living with…, but today it's probably not discussed anymore, because you are able to buy drugs, and people know it's not a death sentence, and you get to move with your life as normally as you could. Right? So, I would say people don't want to get tested or screen for tuberculosis, speaking as an African, I would say the normal one problem it's stigma. Probably the second most closet reason would be funding. Back in Sub-Saharan African Health Care is quite expensive. Good health care. Most people rely on herbs or self-medication to get by. There is probably no… government wide health care coverage, it's the only people who have the essential, that gets what you call GJ move back home that it gets decent health screen. So, people go through 9 month of pregnancy only to give back and hang on medical district. So I would say, probably funding it's the close second.

I want to say that the third reason would probably be, I would say no the lack of information because the information arrives, the writing it's only on the wall, I would say maybe literacy. The government can put as so much posters out there, but if you can't read and you don't understand, what do you do? So, once in a while they get to put out ads in the local (silence…)

Researcher: Did we just lose you there, or you still with us?

P.1.2: It's that my audio?

Researcher: Ok, you are now back.

P.1.2: So, the government try much as possible to put the information out there, but when you are living in a community that it's 60 km from civilization, you miss the poster. You see a guy dress on overalls telling you that you need to vaccinate your kids, and your grandpa it's telling you vaccination don't come and meet my kids never put it. So, I would say literacy would be the third reason why people don't want to be screen for tuberculosis. All those reasons may fall on the categories, are the three major categories. Thank you.

Researcher: Thank you. So, RA2 feel free to chip in case you have some follow up questions. At this point, I like to speak to the stigma that you alluded to. What are the sources of that stigma? Where does it come from?

P.1.2: Stigma it's definitely coming from the society. Stigma it's the society itself. You live in a community where people believe that the man it's the head of the house, and the woman it's meant to work, you live in a society where … Once you come up with something that it's different from the norm you get stigmatized, right? So, stigma can be positive or negative. Can even get stigmatized from making it big in the African community, right? So, once you go out and get an education for yourself, they would say, 'Oh! Look at that guy it's the educated one.' You get stigmatize for that. Later on going through a disease that people don't have or seen before, or they have it but they don't know how to call it and it cannot be treated by the local herbs, so you become an outcast. So, stigma is when you do something or something happen to you that makes you different from the others members of the group and get you isolated.

So once the HIV/AIDS broke out, I keep on going back to HIV/AIDS because it drives African or South Sahara African, malaria use to be the number one killer of infants below the age of 5 back in the '80s, now everybody got used to malaria, right? So, stigma comes from the society. So, I would say, once you become different and you start coughing and everybody looks and say, "What it's wrong with you?" Nobody wants to tell anybody else, "I think I had that disease they are talking about," right? Look at the coronavirus today, 8-months, 9- month down the line, we had sufficient information to keep our self …., but I have one, two, three people that had the virus and people they don't want to tell people that they had the virus, so, it still go down to stigma and we are in North America. So, stigma it's when you get something, or something it's put on you that secludes you or makes you different from the society, and you get isolated from being that person.

RA2: Why do you think they don't want to say they have TB? And I know you are going to say stigma, but I want to develop a little more that concept. If I say I have TB, what happen in your home country?

P.1.2: So today if you say you have TB, somebody is going to take you to the hospital. Back then, if you said you have TB, probably you don't have money to take care of yourself, you don't want to be the guy that's leave out. I can't really please the reasons that anybody would say sorry I don't want to be the one that says 'I have TB,' so, I go back to the three reasons that I mention. You don't want to get stigmatized, lack of funding to take care of you, right? I cannot please the reasons why somebody wants to say, "No, that didn't happen to me, I don't have TB," no.

P.4.2: Can I also say something on stigmatization?

Researcher: Absolutely.

P.4.2: I have an idea just about this stigmatization back from the southwest in general, from back home. When we say one of the line issues is that, we mention funding, we mention people not wanted to go out there, because the feel is they just have common illness, just like cough, by the time they realize it's TB or is a major disease the 4 points of call will not even be at hospital because they don't want to be stigmatize, because people around them feels it's a death sentence. Because if you declare, if you may people know you have TB everybody around you has a possibility to think, if they move close to you, if they talk to you about it, they might be, they might contact.… so they don't want to have, they don't want to come close to even offer you help or to offer advice. People know because of what people might say, what people might think, what people might…, they want to run away, they would rather run away, avoid suppers, all together. You know, we said that delay in diagnosis, would mean delay in treatment and would mean possible death or something. So, if all those stages, that **P.1.2** mentioned, all those 3 things are the major issues of stigmatization. The people, worse than anybody realized, Oh, could be just TB? They would run, keep quiet, they would run to treated themselves, so people don't stigmatize. So, I don't know if am able to say some… There is a way people look at them. There is a way people try to see them, 'Oh, this person it's gone already.' Also, some people wait, they wait until it gets worse. They wait until they eventually land in the hospital. So, it's part of the process, it's part of the mentality, try to avoid stigmatization. If I go out to say, 'Oh this is what I have, they will start running away from me.

People know that the government, vaccines are there. And people they don't make these things on signs. Signs it's a major factor because when you get treatment early, the chances that you get fine is higher. So, when treatment it's late, and the body started to change, maybe the skin, maybe the person it's getting very lean, that it's the time when people start stigmatizing, they would start running. I think stigmatization arise in high level. People that are ill, that are down with Tuberculosis. Those are the pictures I had on my head. When I started going to the hospital, of people that are already looking sick, lean, ice owls, ice eyes inside. So, they look different, and nobody would get closer to that person. So, I don't know if I'm able to post the issue of stigmatization.

Researcher: Thank you for that contribution. P.2.2 what are your experiences around this subject in South America.

P.2.2: You are talking to me?

Researcher: Yes.

P.2.2: Can you repeat again? Sorry.

Researcher: I was just wondering what your experience is in South America around the issue of being screened for TB, problems associated with that, also issues around stigmatization.

P.2.2: About that no, because when I…well, during my childhood we had vaccinations. So, as soon as you are born you get the vaccination, so, in my country I never saw something like that stigmatization. No, no, no, no, I really don't know. I know that because of the crisis we are living now, in the jails, now the prisoners are starting to suffer again. This disease was eliminated, but now it's coming again, and it's starting in jails. But I don't know like, I cannot tell you a deep conversation about that because I don't know exactly. But I know we are having again the sickness and it's because there is no vaccination.

Researcher: Ok.
RA1: Because we are talking about the latent TB, so it's basically like sleeping disease. Do you think that people don't go for screening because they are not suffering? Because there are no signs, no symptoms. Because I am a dentist, I know that people come when they do have pain, if there is no pain they won't come. So, do you think that because there are no signs or symptoms for latent tuberculosis they won't come seeking for screening?

P.2.2: You are talking to me?

Researcher: Not necessarily, any participant.

P.2.2: Like avoiding? Avoiding to get tested?

RA1: Yeah.

P.2.2: I don't know, really don't know. Maybe, maybe they want to avoid it because they know it and maybe they can get rejected from another people. I really don't know. From my experience, I don't know.

P.5.2: One thing I feel is, they might avoid screening because lack of awareness of the severity of the disease. They might just start as a simple cough and practically they might not know the in-depth of severity, so they keep unaware. That might be a cause.

Researcher: Ok.

P.1.2: So, if you say, …(silence) assess it in the body, like it's not pronounced, it's not shown, it's not obvious. That's what you mean by latent.

Researcher: correct, so latent TB is you are infected, but the body somehow is able to contain the infection initially, so during that period you are not able to transmit the disease, but you actually have the infection.

P.1.2: I think probably in that case is because if someone doesn't notice the severity of the disease they are carrying, like RA1 said, nobody wants to show up by the hospital saying, "Hey! I think I have latent TB, check me out." So, most people go to the hospital once they have medical check already pre-planned, or the sickness it is starting to show symptoms. Nobody goes to the hospital just saying, "Oh! I think I may have a recessed form of TB in my system, come screen me." They don't know the severity of what they are carrying. It is potent, but yes, they can't transmit, but they don't care, they don't want to go to the hospital. So, I would say, that would be the reason they don't want to go.

Researcher: Ok, thank you.

RA2: I have another question, going back to what we were talking about the stigma. What happen when the person infected begins to cure, becomes healthy again. Because what you say was when they look sick nobody wants to go close to them, but what happen, when they get treatment, and they get better? The stigma still going on or things change?

P.1.2: I don't think so. I try to put my definition of stigma on an umbrella, saying stigma comes from the society. Once you are different you are isolated, or you are secluded, right? So, in this case, once you are not more different, that if you had got in treatment, you are not more isolated, automatically the stigma goes away. I use my definition backwards. Once you get treatment what stigmatize...

Researcher: Ok. **RA1** would you like us to move on for the next one?

RA1: I think for the third question we were kind of we are answering yes.

Researcher: That's true.

RA1: Why would some people, who had tuberculosis, don't want to tell her friends or neighbours? And I think it's the same like reason that they won't go for screening because again for stigmatization, but if anyone would like to add.
Or if anyone want to elaborate more for the reason why some people want to hide the fact that they are having tuberculosis from their friends or neighbours?

(Silence)

Researcher: Is there any contribution in this regard? Where someone is diagnosed with tuberculosis and for some reason they feel, they don't feel like telling either family members, neighbours about their condition.

P.5.2: I never had a chance to meet a person who had a particular TB, but I'm assuming they might be feeling shy or feeling bad to tell their relatives or friends because recently, we know the covid-19 was detected, and in my home town there was a person who was affected with covid-19, so, I'm assuming they might have the same reasons, they might feel themselves as bad and they got bad disease. That it's just my assumption.

Researcher: Thank you.

P.1.2: Another reason why people might not want to tell their families or friends if they have tuberculosis or any type of diseases that it seems to be a death sentence, is this… how do formulate that? I'll call it Patriarchy. So, what do I mean but that? In Africa people see men likes kings, lions, like warriors. Warriors are human beings too, right? So, when a man it's broke in Africa, they feel they can't put food on the table, they become an outcast, they tend to leave the house, they want a go find money, they want go hunting to bring food to the table. So, this king of Patriarchic kind of behaviour tends to, in some way or the other, emasculates men, and make them feel like they can't do the rules, they can't play the rules of a father, or a brother or a parent anymore. So, once they have tuberculosis they can't go to work, they don't want the appearance to see them as an amusing, quote and non-quote, an inferior man, 'Oh! He is the man that has tuberculosis.' They tent to see you in bar lights. And this is not specifically to tuberculosis, it's just a form of Patriarchic behaviour that wants you as far away from the norm, people treat you as an outcast. So, it's minor from people stigmatizing you, but also people using the African culture, the African tradition believing that men are superior, men are kings, and men are God. So, once you are down, you are sick. You don't want your brother, or your parents, or your fellow countrymen to hear that. You still want to pull the fact that you are a strong, strong African man. So, that tents to play the resend, I don't want my brother to look. You don't want to put that burden on them. You are old, you are sick, and you need to get treatment. So, this goes in long way by, making men like live with sickness for a long period of time. I mean, many men in Africa live with impotency, and they don't tell anybody, they don't get treated, right? For women, it's not so much a burden. Once a woman, and that secluding this to only tuberculosis, once a woman it's different, from tuberculosis or any other form of illness, people in the community tent to encourage the woman find another wife or make a new family. So, we always, the culture always finds the way to by cast orthodox medicine in the form of, or in the guides of culture, in the guides of Patriarchic. Those are the kind of acknowledge that I grew back in the '80s, in the '90s. Now not too much with the advantage of social media, more information in life, women are upgrade in the family, men are more open to be truthful, open to getting help if they need it. That would be another reason why people will not want to tell the family or put the burden on them because of Patriarchic.

RA1: Next question it's actually in two parts. I'll start asking the first one. What would make someone with latent tuberculosis infection, want to take treatment? So, it's latent, there is no signs, there is no symptoms. So, why would someone seek for the treatment?

P.1.2: Acceptance, wanting to get better, more information available. If everybody understand that it is not a death sentence and get better, you tense or foot loon. So, you may get seek appointment, like I don't care what the society says all I want it's to get better, right? The will wanting to stay alive and wanted to stay elderly it is also the reason why someone want to get screen or want to get treatment, right? Sensitization, that is more information available, or accessibility to treatments. If it's vaccine or treatment for tuberculosis is an over-the-counter medication, everybody … but if you need to see a doctor, or if you need to get prescription (silent) I mean, if you need to see a doctor and you don't have medication you can't get it right. If you can walk into a drugstore and get 2,3 or 2,2,2 pills for cure for tuberculosis or the magic tools of tuberculosis, and you can walk to Shoppers or any other drugstore anywhere in Sub-Sahara Africa, if it's accessible, then you don't care, you just go and get treatment. So, that is what I feel.

Researcher: Any other contribution?

P.4.2: Basically, the reason why someone would want to seek for treatment is, first of all, is awareness. Is aware of the possibility of contracting this disease. After awareness, he mentioned accessibility, which is a very shown one, accessibility and not be broken down. Say testing is free, if it's not free, I don't have this disease, but if it's free, why not? Then I just go for the test, I get myself tested. It's free, so, either testing, either treatment, either diagnosis, so is readily accessible, it's readily available, especially the testing. The person is about, "why would I go for testing?' So, I'm aware this testing exists, ok I'm aware, I can afford it. Oh! It's free, then I go for it. Ok, let me get that done, and let me get it out of the way. If it's free, let me just go for it, and I know if I don't have it. And if I get tested because it's free, and I have it, then I get treated, because the treatment is available, maybe the doctors, maybe the hospital. What a view that you need to be tested it's easily and probably free, and probably affordable. Even if it's not free is affordable and accessible. Yeah.

Researcher: Thank you for that contribution. Are there any other contributions before we move on to the second part of the question?

RA1: So, I want to clarify something, when someone is having latent tuberculosis is not suffering, there is no signs, there is no symptoms, is not sweeting, it's not coughing, it's not losing weight, because maybe he is now, or she is healthy, maybe this person is young and his immune system is strong enough to keep the bacteria hiding, to keep the bacteria sleeping, but at some point, when we get older, sometimes we are having a disease and we are getting medication for that specific condition our immune system might get lower, and because of that, this sleeping germ in our body they might get activated and then we will start having active tuberculosis and it is very contagious and dangerous for the people around us. So, if someone knows that is on the latent sleeping phase of the disease, I think everyone would go

for the treatment, so that they won't go for the active stage, and then they have to go for isolation, because they will be contagious. So, we can say it might be prevented to take the treatment.

P.4.2: I don't understand the question.

RA1: I'm not asking a question, I'm just talking about latent tuberculosis. There is a treatment for the latent tuberculosis even though the person is not suffering and there is no signs and symptoms. But just to prevent the sleeping phase develop into an active phase.

Researcher: So, if I may add, what would make someone diagnosed with latent TB not want to take treatment? What would be the motivation behind that? (Silence) Any suggestions. If the health care professionals perform a test on you, and they find that yes you are indeed infected with latent tuberculosis, what would make some people hesitate to be treated for the latent infection?

P.1.2: Two things come to my mind on that question, one is complacency and the other one is poverty, and I am saying this in the context of West African. Complacency, as a human being, out of sight is out of mind. I don't know about… an average human being, once the problem it's out of sight, we tent to push it, to keep it down the road. If it is latent, we say, "Oh! Don't worry." I mean, take a look at the coronavirus today people say, "Oh! It still a hook. I'm not going to wear a mask, just for the fact that is mandatory." That is complacency. People are saying, "Oh! It's just like the flu." That is complacency talking. Once people "Oh! It's not that bad, it's just killing people among 65 years old." That is complacency. Saying that it is latent, it's just like saying, "Oh! Don't worry I'm not going to get it, it's only affecting people in their late 60s." As a human being once we get some kind of relief… Take a look at the province for an instance. We have days where we had no cases, then we went from no cases to travel cases, complacency. That would be one of the reasons why anybody would say, "It's just latent, don't worry about it. I don't need to get screen, I can still go, I will just be careful. I will just watch it, once it just came back, I would fix it."
That is one, secondly…and I say that is Sub-Saharan Africa. People see going to the hospital as a luxury. So, once you tell someone, don't worry you have malaria, all you just have to do is stay away from that mosquito it's going to bite you, so you don't contract the disease. They don't take care of the standards that are around in the environment, they don't use malaria drug, they go to the bushes and get some leaves, they put it on hot water and they start drinking that. So, poverty it's another reason that would make somebody who have hidden, or recessive, or latent tuberculosis in their system does not want to get treatment. Thank you.
Researcher: Would you suggest that those would be some of the motivations even here in North America like Canada in particular?

P.1.2: Complacency yes, poverty definitely not. So, if it had to say the States they have no free Medicare, I don't know, I didn't live in the States, but in Canada no. I mean, everybody has access to health care, right? One way or the other, you will stay get screened. Treated? Yes, we can talk about the budget, what is going to cost you to fix it, but you can still see a

doctor to get tested. In terms on complacency, that is just human nature. Everybody is complacent in one sense or the other. I would say that is a general norm in North America, Sub-Saharan, Africa, or the Asia Pacific, it doesn't matter.

Researcher: I will just add that for our situation in New Brunswick or Canada, in general, the cost of treatment will be covered by the health care system.

P.1.2: That is good to know.

RA2: I have a question. Supposing that P.3.2's father had the latent TB, because he was in contact with another child that died from tuberculosis. Why do you think [P.3.2's name], he didn't get treatment for the latent TB that we suppose he had?

P.2.2: Yes, that is a question that I have. I don't know why, they didn't ask him to put it. Even though when he was tested, he never got it. I was doubting about that, so I asked him again. Because when we were talking about that, I was, "I really don't know." So he told me, and they never asked him to put it. I don't know, but yes, he is not vaccinated my dad, never. And he is really, really healthy. He used to live in a farm when he was a little guy. My grandfather used to have a very big farm with a lots of fruits and a lots of vegetables, he always ate very, very healthy. He had cows and everything. He had a very healthy way of eating the whole time and after my grandfather died. Because, another thing, a snake bit him, and there was no medication for that. He died because it was very poisonous. After that, they moved to another place, they started a new life there, in the north of the country, and they met these people. These people, his friend and her mom, they were not from [Country name], they look like [Country name], but maybe they were from Europe, he was not sure. …That is what he remembered, it was long, long ago. As I told you my father is already 81, 82 years old. They were immigrants, they were not from [Country name], but they don't know if he got the illness in the country, or they brought it from the place they came from. He never got the vaccine; I don't know why.

RA2: So, they didn't give him the shot, and they didn't tell him about the possibility of having latent TB. So, it's lack of knowledge.

P.2.2: Exactly. Nobody explained to him like what you are doing about that. They say, "No, he is right, his immune system is so good, he is protected," but they never thought, I think, he had the bacteria inside him. Yeah. And now I think, because of this, that maybe he has it, but he is healthy.

Researcher: All right, thank you for that contribution.

P.2.2: You're Welcome.

Researcher: RA1…

RA1: That bring us to the last question. Anyone would like to add anything about latent tuberculosis before we end the discussion?
Researcher: So, there are any last minute contribution anyone would like to make before we end the discussion? (Short Silence)

Focus Group Discussion #3

Participants: P.1.3 (South America), **P.2.3** (Asia), **P.3.3** (Africa), **P.4.3** (Africa)

RA1: Thank you everybody, I will start recording and start asking the questions. Let's make this like a discussion, and not like questions and answers. Is more kind of discussion. So, our first question, I want for you to tell me about your experience with tuberculosis in your home country. So, here we are talking about general tuberculosis. What did you hear about tuberculosis back in your country of origin and also, we would like to know if you have any experience here in Canada after you moved.

P.1.3: I never experience been close to a person with…(rumbles and have an echo… (inaudible)

Researcher: So, if I heard you right, in your family you didn't experience anything around tuberculosis. However, there was someone in your neighbourhood, there was a child in your neighbourhood who had tuberculosis. Is that correct?

P.1.3: Yes.

Researcher: Are other experiences with tuberculosis in your home country before moving to Canada?

P.3.3: I had an experience with tuberculosis with my sister before coming to Canada. That was 15 years ago. We had to do medical examination before coming to Canada and she tested positive. We had to stay back there, and had a treatment before coming, but it was only her that tested positive, no one else in the household tested positive.

Researcher: So, tell us about the experience had with your sister.

P.3.3: Well, like I said, we didn't know that she had it till she had a cough, she was coughing, she spat out some blood. We went to the hospital, check her out and they said she was positive of TB, so the entire household has to be tested as well and after we were all negative. She had to stay in the hospital, and she was isolated for about 2 weeks getting treatment. We were not allowed to visit her while she was in the hospital because she was on isolation, but it was only for 2 weeks on treatment. The thing with TB was that the nurses and the doctors assured us that the germ can be kill it's not life threatening, so she was going to be ok and come back home. So, that was good for us.

Researcher: So, good to hear that she was able to be treated, but how did the other members of the family or the community take the message that your sister had tuberculosis?

P.3.3: It was scary, people don't want to come to visit us because of that. You mean after she was better, and she came home? It was something like they can get the virus, so they didn't come much. It was a long time before people gradually came around.

Researcher: Are there any other experiences from other participants?

P.2.3: Hi, back in my country I haven't heard of this. Actually, I heard that people had tuberculosis many, many years back. But we haven't gotten into contact often right now. For us it was some disease that was in the history, I really didn't know about this. Hearing this, I feel ignorant about that. That this is a virus that get spread like that. I didn't know. After hearing about it, I was really shocked that this is something like a covid, I didn't know anything about that. So, this is a completely new news for me. And after coming here also I haven't heard about this thing, only through the survey.

Researcher: Ok. So, tuberculosis is caused by a bacterium, not a virus. Just I thought I clarify that.

P.2.3: Ok. I didn't know that. A common blind vision had to be very careful about that posting, I was ignorant about that. That's what I am saying.

P.4.3: For me too. I had an experience with my mother when I was among 5, maybe 20 years or something. She had TB. But in our country, everybody takes the vaccine. Babies after birth to 2 months. I call it a scary experience. She got isolated in our home for a month and a half. We, also, as a family, she was isolated from us, but not too much because we all had the vaccine. You can't go to any school if you didn't take the vaccine. But it was a scary experience. We check that everyone had the vaccine so nobody will… It disappeared from our society for a long time, three years to find someone to have TB. That's it.

Researcher: So, a quick question to you. How was your experience with your mom and everyone else in your community when your mom had tuberculosis? How did everyone else treat your mom or treat everyone in your family?

P.4.3: For my mom, she was isolated, so no visits. Even though for us we had no contact with her. But for us was…we went to school normally, my father was going to his bedroom, also, normally. We acknowledge about this disease in our country but a little bit relief that everybody has the vaccine. So, it you will be contact with her that is the thing that would be dangerous, but if you are not being with her, it's ok. We didn't get somebody to care of us, nothing like that.

Researcher: Ok

RA1: That was for general tuberculosis, we would start to talk about something more specific.

RA2: Can I make a comment before you continue? Because from what I hear from P.4.3, she believes that because she had the vaccine she is protected. Isn't it?

Researcher: It's that correct P.4.3?

P.4.3: Yeah, because you take the vaccine to be protect from that disease.

Researcher: Ok, we can talk about that a little bit more later.

RA1: So far as I said we talked about tuberculosis in general, now I want to introduce something to be more specific, we are taking about latent tuberculosis. When we say latent tuberculosis, we mean that is like actually sleeping in the body, so this people are not having signs or symptoms, so they are not suffering, no coughing, there is no sweeting, there are no losing any weight, they are completely as normal as the other people without latent tuberculosis. So, I would like to ask, why some people would not want to get screened for tuberculosis?

P.1.3: (Inaudible) I can say that probably …because they are afraid… that people get close to them.

Researcher: Why do you think they would be thinking like people, maybe they are friends or family members, and don't want to get close to them again?

P.1.3: Yes, because, by knowing how the disease affect to people, they will be…, they will think that their life is in danger about getting the disease.

Researcher: Ok. Are there any other contributions from other participants?

P.3.3: Just to add to what he said, people are afraid to do the test because they might feel that they'll lose friends and family, or they might be neglected, and also some people just don't know what the bacteria is all about. Some people had been heard about it, so they don't know, they don't have any education about it, they don't know about it, and stuff like that. So, that would be something that they might not want to be tested, because they don't know what the bacteria is about, or what it does to people.

Researcher: So, that sounds like there maybe are in need for some education there.

P.3.3: That's correct.

Researcher: Any other contribution on this question?

P.2.3: I'm also in the line that education should be collected because many of them, may not be knowing about the disease. I might should tell we all are cover with the vaccine, that is mandatory to take it now in our country, and that's all.

Researcher: So, something about vaccination for tuberculosis needs clarification in that yes, indeed most of our countries of origin do take the opportunity to vaccinate, particularly children, but that vaccination, that protection is not necessarily lifelong. Along the way, you may still be able to contract tuberculosis, but it does a fairly good job in preventing the younger children from getting tuberculosis.

RA1: So, I think that would be new information for P.4.3 because she mentioned that the whole family, the whole community are protected because of the vaccine.

Researcher: Right. Maybe we can move on RA1.

RA1: Our third question is, why would some people who had tuberculosis not want to tell their friends or neighbours?

P.1.3: (Inaudible) Do you hear me there?

Researcher: It is a little bit hard to hear you, sometimes it is clear, other times it's cutting.

P.1.3: As I was saying…. I consider that people will do keep it in secret because they don't like to be like… and other people not knowing the consequences of the symptoms, they would try to keep as protected as …that including the family, and everybody surrounded.

Researcher: So, where does this issue of the fear or exclusion come from?

P.1.3: Sometimes when people know there is a disease that can affect your body and your health, in the way that tuberculosis affects, they try to keep away. May people I know, in my country, they believe is a disease that can't be cure, also not having the resources to get medical assistant. That's why they want to be protected from someone infected. I know that this respiratory disease (inaudible).

Researcher: Ok, why don't we wait for other participant to make their contributions? So, there are two situations. Situations in our countries of origin, and now here in Canada, in New Brunswick in particular. Is this something you can feel free to tell your friends or neighbours about; that you have been diagnosed with either latent TB or active TB? Because in terms of the danger, as you said in our countries of origin maybe, the health care system was not as good, or maybe the medications weren't there, but whereas here in Canada the health care system will pay for the medication. So, with that information, would that make someone feel more free to talk about the diagnosis of TB here in Canada.

P.3.3: I think so.

Researcher: Would you like to elaborate?

P.3.3: Well, just from experience, some of our countries, the mentality behind setting illnesses, when you have for example if you had AIDS, people would look at you differently, they will be putting you in a different classification. Like, "Oh! maybe this person is a bad person, or they have sex everywhere they go," and stuff like that. So, if someone has AIDS, they won't discuss that with everyone, the same as tuberculosis. When you have AIDS, in some cultures, they think you are bad, you do this, you do stuff like that. So, both illnesses people don't want to talk about it. But in advance countries, like Canada, they have treatments. I don't think that people are mostly judge for what kind of sickness they have. So, coming to Canada and the experience I had back home, there are two different societies. I don't think you will be judge more like advance country, like Canada compared to the cultures we come from where, it's a developing country, they don't have proper health care, they don't have medication, sometimes it's expensive to get treatment, so some people just stay home and maybe self- medicate themselves. Yeah.

Researcher: Thank you for your contribution. Are there any other contributions?

P.2.3: The experience from the country I am coming from, is that people are aware that they will get good medications, but the expensive ones made a problem there. So, whoever cannot afford that, might not be going for that, in the initial state itself, they will try to delay that. So, that would be a blandish for people coming from my country to Canada because we are more confident that expense will not give us impress, so I think people will go and get treated. Thank you.

Researcher: Ok, thank you for that contribution. Does anyone else have anything to say on this topic?
Silence.

Researcher: Ok, so, looks like we may have to move on to the next question.

RA1: It's kind of we were answering next question because it is interconnected actually. Our first question has two parts, I'll start asking the first one. What would make someone with latent tuberculosis infection want to take treatment? So, we said that they are not signs, no symptoms, but what would make someone, with a latent tuberculosis, go and seek for the treatment?

P.1.3: Can you repeat?

RA1: Yes, sure. What would make someone that is diagnose with latent tuberculosis take the treatment? Like, this person has a latent tuberculosis and he or she is not suffering from signs or symptoms. But why do you think that this people go and take the medication?

P.1.3: (Inaudible)

RA2: P.1.3, hold on a minute, please. Researcher, if P.1.3 talks, can you repeat? Because I think you are the one that can hear better. The recorders are not getting what he is saying. If you can repeat what he says it would be awesome.

Researcher: Ok. Yeah.

RA2: Thank you. P.1.3, go ahead.

P.1.3: (Inaudible)

Researcher: All right, so P.1.3 is saying that the situation where someone diagnosed with latent TB would be encouraged to go seek treatment in order to protect others, like his or her love ones, or family members and so on. Knowing that if you don't get treated then you may eventually suffer from the disease and potentially spread it knowing the severity of the disease when it actually happens.

P.1.3: Yes.

P.2.3: I agree with that, is the person diagnosed with latent tuberculosis is properly inform with that, he can get through this, and he can stop spreading and he can help his family as well. So, he will surely come and take the treatment, and be initiated with that. But he should be informed that what he is missing so know and he can get to all those things very easily, then I think he will get the treatment.

Researcher: All right thank you so much. Just to reiterate that in latent tuberculosis stage, you are infected, but you have zero symptoms. The problem starts when you have active TB, so, that is when the symptoms come, that is when the cough starts, that's when the sweeting and so on.

P.2.3: Yeah. If the person is not having any symptoms, he might be feeling that there is something maybe wrong or the new perfectly fit. I think he should be well informed that what is the consequence after. That is what I think.

Researcher: Yes, absolutely. Thank you for that contribution. Was that P.4.3?

P.2.3: P.2.3.

Researcher: P.4.3 any contribution on this subject?

P.4.3: No, actually no.

Researcher: Ok. Silence.
If we bring it closer to home and say if one of us was diagnosed with latent tuberculosis here in Canada. If you go to see your family doctor, and among the test that they conduct they find

that you are actually have latent tuberculosis, is that something you will seek treatment for? And if yes, why? And if it's not, why not?

P.1.3: In my opinion I will look for treatment, considering we want to keep the society in a healthy condition.

Researcher: ok, so, P.1.3 says that he would be happy to seek treatment knowing that will help keep everyone healthy, including the rest of the community where he is living.

RA1: I think P.3.3 wanted to say too…

P.3.3: Just to add to what P.1.3 just said, if you are diagnosed with latent TB, you need treatment because eventually going to become active, so you need to treat it before it gets to that point.

Researcher: So, the chance, if you are not treated for latent TB, you may have a higher chance of developing active TB down the road. If it is treated, you significantly reduce the chances of ever progressing for latent TB to active TB.

P.3.3: Yes.

RA1: Thank you everyone for your participation. Now part 2. Why would you think some people, when they are diagnosed with latent TB, they don't want to get the treatment? So, we are here in Canada, we said that we have a good health care system, the treatment is cover, and there is no financial issues. Why would you think some people won't go for treatment?

P.1.3: Maybe because they don't know for sure, how the disease is treated. That would be a possibility. That they are afraid of having some kind of medication that probably affect their body. I can see that. Knowing that we are in a develop country, I know for sure that people will be interesting and very optimistic to get that treatment.

Researcher: Where you able to get that, RA2 on the recorder or you want me to repeat?

RA2: It would be better if you repeat. Can you repeat, please?

Researcher: Ok, if I can remember what he said. (Laughs). Basically, he was saying, people would feel encouraged to get treated here in Canada, knowing that some of the barriers associated with difficulty to get treated for tuberculosis have been overcome, like the cost of treatment has been covered. It appears that is one way in which someone would feel more confident knowing that the available health resources are there.
If you wanted to make a further contribution, please go ahead, and then maybe…I'm not too sure if I captured what you said correctly.

RA1: I think there was one more part that P.1.3 mentioned. Like some people, they don't want to take medication that they might think it might affect their body in a certain way, so they want to avoid taking this medication.

Researcher: Absolutely. I think you alluded to the fact that maybe the side effects of the medication. People don't want to experience any of those. But, if you think about something like that when people are generally younger, those are things that the body can tolerate more, compared to when they are older and maybe they develop active TB then, because their body system is weaker. And then, if you have active TB you have to be treated with so many drugs. If you have latent TB, the treatment is only one drug. So, there is a big advantage in getting treated when the infection is latent as suppose when it becomes active TB. And when it becomes active, then there is a danger of spreading it to other people. Technically, if you think about it, if you are treated with one drug, it is also cheaper compared to treating with 5 or more drugs.

P.2.1: I'd like to add, because of the country from I come, there are different types of medication are not showing there. I am from [Country name]. People tend to go to take different varieties of medication they can, a well or pre-hand. Traditional form of treatment they finally come to be taking drugs or the pills as self-medication, so, that might be a problem because initially when they are having a latent tuberculosis they might not be coming for the treatment, and they are making it severe or getting severe, they will be reaching the hospital.

Researcher: So, there is a health seeking behaviour issue there. Where some people either just don't want to go to the hospital quickly because maybe are thinking it's just a simple cough or they try traditional medicine, and if it doesn't work, that is when…the last resource is going to the hospital.

P.2.3: I think that might not be the case in Canada, because here the medication is free, so people might consider the family clinic and all. I think that would be the case here.

Researcher: Are there any other contributions from other participants? P.4.3, I get to you at this time. Any contribution?
No response.

Researcher: Ok, I guess not. What about P.3.3, do you have anything to contribute to this question?

P.3.3: You have a question for me?

Researcher: What do you think? What are your thoughts?

P.3.3: My thoughts about the topic of tuberculosis is, the awareness needs to go out there, people need to know what the TB is all about and the effect of it. I think, coming to Canada,

with the advance health care they have here, it's not common that people have TB in Canada, so you tent to find that people don't know about it much. You don't hear people talking about it here in Canada, but where I am from it's pretty much like people do talk about it and is everywhere in the community. So, the awareness needs to be out there in the community in Canada, even though I never meet anyone in Canada with TB, but people talk about it. I was surprised when I got the call from Caro the other day asking me if I wanted to participate in this research. I'm like, I didn't hear about this in 10 years, 10 years before. So, people need to be aware of it.

Researcher: Right, ok. All right, thank you for that contribution.

RA1: Anyone would like to add or share about latent tuberculosis infection?

P.1.3: I think that in countries like Canada, they don't experience this disease as common as other countries, like ours. The issues have some king of educational, kind of documentation, so people know more about it and know where to go, in case they have, they feel symptoms. From my personal experience of travelling here we had some exam done before to check if we have any disease like this one.

Researcher: Just give me a second. This contribution includes the fact that in various countries outside Canada, where TB situation is more than, more cases of TB, there is a little bit more awareness. Here in Canada, there isn't that awareness a lot. So, there is a need of provide some level of awareness, particularly with reference to some documentation about tuberculosis, and if you have it, this is what you do or this is where you go to get it sorted out. Sorry, I interrupted, were you going to make a contribution about your own personal experience?

P.1.3: Yes. Knowing that you are going to travel to another country, you want to be… (Inaudible)

Researcher: So, the contribution was that, knowing that you are moving over here from which ever country, then there is that need or that feeling that your health needs to be as good as it can. In order for that to happen, some level of information need to be there, so that somebody is well aware of what needs to be done. On that note though, you did allude to the fact that there were some tests done when you are moving to Canada. Did they tell you exactly what those tests were? Would you like to speak to some of those?

P.1.3: You have an interview with the Doctor, we can call it the family doctor, the one that assist you in all the exams and all the tests. They share with you all of the results and then they send it to immigration.
