# Supplementary material for: Tuberculosis related barriers and facilitators among immigrants in Atlantic Canada: A qualitative study
Source: PLOS Glob Public Health. 2023 Jun 5;3(6):e0001997. doi: 10.1371/journal.pgph.0001997 (PMC10241363; doi:10.1371/journal.pgph.0001997)
Supplement: Attachment — Submitted filename: Response to Reviewers.docx [file pgph.0001997.s002.docx]

**Responses to Reviewers’ comments**

| **Reviewer’s comment** | **Authors’ response** |
| --- | --- |
| **Reviewer #1:** The authors have clearly summarized the perceptions of recent immigrants to Canada about TB prevention and care services. Findings are in line with the literature. |  |
| Please review the COREQ checklist and include missing items in the manuscript's methods section. | - Addressed by adding adding a statement in lines 185-186 “To ensure reporting quality of our study, we relied upon the Consolidated Criteria for Reporting Qualitative Research (COREQ) [73].” Also, a COREQ checklist is attached to this document. |
| **Reviewer #2:** Thank you for the opportunity to review this manuscript. It adds new information to an important topic. My comments can be found below: |  |
| **Background** |  |
| The background section should provide more context regarding the number of people with TB and LTBI diagnosed each year. | - Addressed by adding (lines 93-96 ) “Currently, Canada has one of the lowest active TB rates in the world [10]. In 2020, Canada reported 1,772 active TB cases of whom 1303 (75.6%) were foreign born individuals. However, it is estimated over 1.5 million LTBI infections affect the Canadian population [27, 28].” |
| Of the ~7,500 immigrants to New Brunswick each year, how many are currently screened for LTBI? | - Addressed by adding (line 110-112) “Currently, there is no routine LTBI screening in New Brunswick and immigrants are only screened for LTBI if they are flagged for medical surveillance based on chest x-ray results or other notable signs. Such is the case in all provinces and territories of Canada [11].” |
| The scale of the TB problem is unclear. | - The following statement has been added to provide context to elucidate the current state of active TB and LTBI in Canada and screening practices in the province of New Brunswick and Canada. (lines 93-96) “Currently, Canada has one of the lowest active TB rates in the world [10]. In 2020, Canada reported 1,772 active TB cases of whom 1303 (75.6%) were foreign born individuals. However, it is estimated over 1.5 million LTBI infections affect the Canadian population [27, 28].” |
| The manuscript should provide more information about the structure of the health system in New Brunswick, health financing model and the cost of accessing TB care. The manuscript does not make it clear in the background whether immigrants would be asked to pay for care out of pocket. Do all of these immigrants have access to health insurance in Canada? | - Addressed on lines 138-144 with the addition of “In Canada, financial burden is not typically identified as a barrier as refugee immigrants are immediately covered by public health insurance under the *Interim Federal Health Program (IFHP)* that covers a wide variety of health care services such as immigration medical exams, treatments, vaccinations, including TB testing and treatment. In the case of economic immigrants there is a three month waiting period from the time of their arrival for their public health insurance, i.e., Medicare, to be activated bestowing the same benefits as IFHP [61].” |
| What about undocumented immigrants? | - Added on line 176, “documented” immigrants - Added on line 466-468 “As immigration around the world continues to increase research focusing on undocumented immigrants will also need to be addressed. |
| Adding this information will help the reader understand when participants discuss affordability of care (e.g. Lines 281- 282) when you wrote, "Individuals do not have sufficient knowledge about the complex healthcare system in New Brunswick..." Currently, the reader is left in the dark about the structure of the healthcare system and what makes it complex or challenging to navigate. | Addressed by editing line 342-345 to now read “Individuals do not have sufficient knowledge about the intricacies of the healthcare system in New Brunswick, such as accessing free short term public health insurance for some immigrant categories e.g., refugees, creating another barrier to receiving care.” |
| One of the main findings of this paper is that immigrants need more education (Lines 395-396) about TB and LTBI, but it is unclear what education and outreach is provided in the region. | - Addressed on line 410-412 added “Currently, there are no educational interventions targeting TB knowledge available for either immigrants or the general public in Atlantic Canada. When questions concerning the development of future educational interventions were asked,...” |
| Lines 116- 119. Facilitators of healthcare also include person-centered policies, accessibility of health financing, etc. Additional literature should be cited here. | - Addressed on lines 151-157 added “Other facilitators for accessing health care services include avoiding stigma, wanting to protect loved ones from infection, having the right to stay in the country, and wanting to know one’s health status [63]. Person-centered delivery of services and health policies that treat each immigrant as a person rather than a population optimize the care of migrant patients [64]. Ensuring financial resources such as health care coverage are consistently cited as important predictors of approaching health care services [65,66] and adhering to treatment [67].” |
| The background is solely focused on Canada, when other high-income countries are also adopting similar strategies to address LTBI. To increase the applicability of the manuscript to other contexts, it would be helpful to include some additional background information on the various LTBI screening strategies and policies being employed in other contexts. | - Addressed starting on line 74-91 with a new section entitled “Screening for active TB and LTBI” - “The World Health Organization (WHO) estimates that one in four people are infected with *M. tuberculosis* and the disease is estimated to have killed about 1.6 million people in 2021 worldwide [10]. This staggering number is unknown to most within developed countries, where TB is not a major burden on their healthcare system [11-13]. The majority of active TB cases in low TB incidence countries are foreign born individuals [14]. Historically, high-income low-incidence countries have managed migrant TB risks by focusing on early identification of active cases, treatment, and contact tracing for close contacts [15-17]. Conversely, the alternate strategy of selectively screening immigrants from high-burden TB countries for LTBI and offering treatment has been identified as being problematic due to implementation pitfalls and unfinished treatment regimens [18]. However, a recent meta-analysis examining the effectiveness of LTBI screening and treatment programs reported an improvement in completion of treatment regimens for LTBI [19]. For example, a voluntary LTBI screening program for foreign born individuals in Sweden has shown low attrition rates in treatment and an overall cost effectiveness in curbing the costs of potential active TB cases [20]. Other studies have also provided the cost-benefit analysis in favour of LTBI screenings and treatment [21-23], however, many countries continue to only screen for active TB [24] and details on who, when and where to screen remain uncertain [21, 25, 26].” |
| Lines 96-104. There is a long history of stigma and discrimination for people with TB and LTBI, yet this paragraph focuses on potential scapegoating similar to that seen during the early days of the COVID-19 pandemic. If this scapegoating of immigrant communities happened in previous TB outbreaks in Canada, then a more concrete example should be referenced, rather than speculation about future scapegoating. | - Addressed beginning on line 122-129 “The well documented negative impacts of TB related prejudicial attitudes in both high burden TB countries [37-40], and low incidence countries [41-46] consistently reveal the relationship between stigma and reticence in TB testing, delays in initiating treatment, low adherence to treatment regimes, and individuals with long lasting internalized self-stigma [47, 48]. This increase in prejudicial attitudes within the community could in turn worsen long-term health outcomes for the entire community, not just the targeted individuals, due to this increase in prejudicial sentiments toward immigrants [49].” |
| **Methods** |  |
| Lines 140-141. The sampling structure should be clarified. It was unclear what "demonstrated high knowledge of TB in the larger study" means. Was this demonstrated through a survey or interactions with study staff? This is an important clarification because the discussion section ( Lines 327-338) seems to identify that the participants did not in fact have "high knowledge." Did the sampling method produce a group that was able to richly discuss the topic? If the participants were not able to do this, then how can the researchers claim that "sampling adequacy" (Line 193) was obtained. | - Addressed in line 176-180 sentence edited to clarify “The participants were purposively invited via email because they either 1) came from countries with a high TB burden, or during the initial semi-structured interviews 2) they demonstrated relatively high knowledge of TB compared to others in the larger study, or 3) they had experience with an active TB case in their community or family, and were thus likely to provide rich insights [72].” |
| Further questions about sampling. Does the high education level of the participants match the overall immigration profile of Canadian immigrants or were higher-than-average educated participants purposively sampled? | - Addressed on line 263-265 “ This high level of education is characteristic of the broader Canadian immigrant population that account for over half of the graduate degrees in the country [81].” |
| Were any of the participants related? | - Addressed in lines 266-267 “All participants were strangers to each other, except one married couple.” |
| In the FGDs, five questions were asked by the researchers, and none specified anything about Canada or New Brunswick. It is unclear why the decision was made to collect information about TB in both home countries and Canada to then draw conclusions only about Canada. | - Addressed in lines 203-205 adding “The progression of questions beginning with their home country was purposeful as it was intended to serve as an easier entry, i.e., familiar, an ice breaker, into what could be a difficult topic.” - Added to discussion in lines 440-445 “It should be noted that feedback from participants in the focus groups was based primarily on their experience in their homeland; however, these experiences contributed to their current attitudes that continue to influence their behaviour even now living in Canada. Further, by including their homeland in the questions, the bulk of their experience rather than their limited time here in Canada was included in the conversation; conversely, by focusing only on their Canadian experience valuable information might have been excluded.” |
| Line 163. Did conducting the interviews in English cause any challenges? | - No it did not. This has been addressed in lines 189-191 “All participants had adequate English language conversational skills, hence, no translators were used; notwithstanding, a translator was available for all focus group discussions.” |
| Lines 191- 200. The section on rigor should explicitly state the methodological orientation of the study, e.g. grounded theory, discourse analysis, content analysis, etc. | - Addressed in lines 183-185 adding “The methodological orientation that guided data collection was a phenomenological perspective seeking to understand the participants’ lived experience and knowledge of LTBI testing and treatment.” |
| This manuscript would generally benefit from reviewing and incorporating the COREQ. | - Addressed by adding adding a statement in lines 185-186 “To ensure reporting quality of our study, we relied upon the Consolidated Criteria for Reporting Qualitative Research (COREQ) [73].” And also by Completing the COREQ and attaching it to the reviewers comments. Note: Transcripts were not returned to participants because the transcripts were verified against the audio recorded interviews (item 23). |
| Table 1. How many participants had direct experience with TB treatment? The descriptive statistics should include previous history of TB/LTBI treatment, or living in the household of someone with TB, if available. | - We did not specifically ask these questions to participants. The only information available is that which was volunteered by participants during the interview, anyone who had any prior experience with TB indicated during the focus group discussion. |
| **Results** |  |
| Theme three is about the barriers and facilitators of LTBI testing. However, this essentially restates your research question. A theme should not be this close to the research question; it should elucidate it. Authors should consider revising this theme entirely. | - Addressed by having coders review transcripts and consult on name of third theme. And if you agree), it was agreed that fear of testing, treatment and healthcare system clustered around one part of the older theme but a fourth theme, complacency, needed to be identified as its own theme. - Hence changes reflecting this new name for theme three and fourth theme are noted on: - line 269-271 “Four themes in relationship to barriers to testing and treatment emerged from the FGDs: 1) need for education, 2) stigma, 3) fear of testing, treatment, and the healthcare system, and 4) complacency.” and, - line 323 new subheadings “Fear of testing, treatment and healthcare system” and, - “Complacency” on line 350, - The abstract was also updated to reflect these changes on line 37-42 “Four themes were identified from the data relating to barriers to testing and treatment of LTBI: 1) Opportunity for education, 2) stigma, 3) fear of testing, treatment, and healthcare system, and 4) complacency. Results included reasons individuals would not receive TB testing, treatment, or seek help, as well as facilitators to testing and treatment. These findings may inform the implemention of an LTBI screening program in Atlantic Canada and more broadly acresss the country.” |
| Lines 215- 225. This section seems to indicate that FGDs devolved into a question-and-answer session, where the expert never ceded their position of authority to the participants. More information should be provided about the decision to select a TB specialist as the primary interviewer and the information that the participants were provided about the researcher. | - Thank you for this comment, to clarify the TB specialist was not a primary interviewer and was only drawn into the focus group discussion when questions that could not be answered by the RAs occurred. This clarification is added on line 195-198 “Focus groups were facilitated by two research assistants, with a senior member of the research team present in case there were questions they could not answer. This ensured maximal information, optimal benefits, and all opportunities for empowering participants. ” |
| Lastly, the manuscript should provide a reflexivity statement that acknowledges and discusses the researcher’s role in the qualitative interview process. | - This comment has been addressed in our response to the prior concern hence a refelexivity statement here is not warranted (see lines 195-198). |
| Line 238. Remove the contraction, unless it is supposed to be part of the quote. | - Contraction removed, line 299, “won’t” changed to “will not” |
| Table 2: At times the theme is written as "TB testing (timing)" and other times "Timing of testing". If this is one theme, then label consistently, or clarify the differences. | - Addressed inconsistency – now reads TB testing(timing) throughout Table 2, other inconsistencies/formatting in this column also addressed. |
| In the results the participants are labeled P dot x dot x (P.3.1) In this table, they are labeled as Px dot x (P4.1). Please use consistently throughout. | - Addressed – thank you. |
| 60% of questions were asked by P5.1. Two more (20%) were asked by P4.1. Did these two participants dominate the Q&A session? Or did all participants ask questions? | - No, not at all, P.5.1 and P.4.1 did not dominate the discussion, there were multiple exchanges with all other participants, however they were the two participants that did ask questions.   Added in lines 274-276. “There was an extended discussion in all three focus groups involving all participants (n=14) in varying degrees, however, only four of the participants asked questions (Table 2).” |
| The Stop TB Partnership, the UN-agency dedicated to TB, has issued a TB Stigma Assessment Tool. This tool defines various forms of TB stigma including: anticipated stigma, self-stigma, enacted stigma, secondary TB stigma, perceived TB stigma, structural stigma, etc. This manuscript would benefit from a more nuanced discussion of the concept of TB stigma, which is already in wide circulation among the TB community. | - This is an important point and perhaps would be best addressed by collecting relevant data asking this research question, i.e., what types of TB stigma exists in Atlantic Canada and where is it specifically happening, e.g., community centers. We will reference this concern as a direction for future research in lines 452-456 “Further, understanding what types of stigmas, e.g., enacted stigma, immigrants to Atlantic Canada experience [96-98] and the specific locales where this occurs would provide insight into how these factors influences quality of care and offer direction on developing local stigma reduction interventions [99,100].” |
| Lines 263- 267. Please clarify this sentence. It is confusing with multiple uses of e.g. | - Addressed with rewriting/addition to lines 324-328 “Several barriers to testing and treatment were identified during the FGDs. Lack of trust and understanding of Canada’s healthcare system, perceptions of personal costs of time and money needed for testing and treatment, fear of treatment side effects, uncertainty if they have the mental resources and social support to cope with a positive test, and trying traditional treatments before visiting the hospital as a last resort, were all reported.” |
| Section on Barriers to testing and treatment. Why focus the results on barriers in both home countries and Canada, and then the discussion is limited to best practices in New Brunswick to reduce health inequities? There seems to be inconsistency between the results and findings. | - Addressed in lines 440-445 “It should be noted that feedback from participants in the focus groups was based primarily on their experience in their homeland; however, these experiences contributed to their current attitudes that continue to influence their behaviour even now living in Canada. Further, by including their homeland in the questions, the bulk of their experience rather than their limited time here in Canada was included in the conversation; conversely, by focusing only on their Canadian experience valuable information might have been excluded.” |
| Secondly, the barriers focus on "socioemotional cognitive barriers" without addressing any structural barriers in Canada. | - Added to lines 464-466 “The influence of structural barriers, e.g., laboratory services, and their relationship with identified socio-cognitive barriers, e.g., stigma, need to be understood in the local context.” |
| Lines 306- 312. This should be included in the results and not the discussion. | - Thank you, moved to results section (see lines 252-258). |
| Lines 352-354. "...culling selected information based on a misinformed confirmation bias." Consider revising sentence to place less blame for a lack of information on people with TB. | - Sentence rewritten, line 406-409, “It is important patients receive accurate information from reliable sources, such as health care practitioners, rather than searching the internet for pertinent health information that may not be pertinent or accurate [89, 90].” |
| Lines 376-378. It may be beneficial to reference the work done in the U.S. by Amera Khan on screening for LTBI among US-bound immigrants here, along with examples from other high-income, low-burden TB countries. | - Added in lines 433-436 “Likewise, Khan and colleagues have found similar success in offering voluntary screening and treatment programs prior to emigration. They had an 88% treatment completion rate for the 21% who tested positive, before arriving in the United States of America [94, 95].” |
| The discussion focuses on "strategies and interventions" for LTBI but leaves out immigration policy change. Consider adding policy recommendations. | Addressed in lines 473-477 “Based on the current study, the following policy recommendations are suggested:   1) The government should promote more aggressive LTBI screening in certain populations. 2) the government should develop TB and LTBI educational programs for newcomers and healthcare providers and ensure a viable work force is in place to manage both LTBI and active TB cases.“ |
| The manuscript describes the geographic coverage as "Atlantic Canada" and "New Brunswick" but then in the abstract and conclusion, it is described as relevant for "southern New Brunswick". Consistency – | - Thank you, inconsistencies rectified throughout manuscript. |
| Please clarify the applicability of the findings. Are the authors making recommendations only for one region of Canada? Could recommendations be made applicable to all of Canada? | - Added in line 457-472 “The current study underscores the necessity for understanding global issues through a local lens [101]. Canada does not have a national LTBI screening strategy for immigrants; thus, this project addresses both a clinical gap in public health and increases understanding of the stigma, fear, and ignorance that may interfere with immigrants accessing healthcare services in Canada. The need for an education program to increase TB knowledge is clear. What is not clear and warrants future attention is if this need for education exists in the broader community. Further, interventions to help immigrants overcome fear of testing, treatment and the health care system are needed to be developed and delivered for the local population. The influence of structural barriers, e.g., laboratory services, and their relationship with identified socio-cognitive barriers, e.g., stigma, need to be understood in the local context. As immigration around the world continues to increase research focusing on undocumented immigrants will also need to be addressed. The findings of this study pertain directly to Atlantic Canada and with future study and time may also show applicability to the rest of our vast and diverse country. Future research based on these findings need to remain sensitive to local immigration characteristics and trends, so by attending to this emerging evidence a helpful response may be mounted to address the local, and perhaps global, need of the migrant [102].” |
| **Conclusion** |  |
| The manuscript calls for more education among immigrants, but leaves out healthcare workers, the general population, etc. Immigrant communities are not the only source of TB stigma, and an effective anti-TB stigma intervention TB stigma will certainly need to address multiple forms/loci of stigma. | - Adding line 452-456 “Further, understanding what types of stigmas, e.g., enacted stigma, immigrants to Atlantic Canada experience [96-98] and the specific locales where this occurs would provide insight into how these factors influences quality of care and offer direction on developing local stigma reduction interventions [99,100].” - Adding line 461-468 “The need for an education program to increase TB knowledge is clear. What is not clear and warrants future attention is if this need for education exists in the broader community. Further, interventions to help immigrants overcome fear of testing, treatment and the health care system are needed to be developed and delivered for the local population. The influence of structural barriers, e.g., laboratory services, and their relationship with identified socio-cognitive barriers, e.g., stigma, need to be understood in the local context. As immigration around the world continues to increase research focusing on undocumented immigrants will also need to be addressed.” |

**COREQ (COnsolidated criteria for REporting Qualitative research) Checklist**

Adapted from: Tong A, Sainsbury P, Craig J. Consolidated criteria for reporting qualitative research (COREQ): a 32-item checklist for interviews and focus groups. Int J Qual Health Care. 2007;19(6):349-57. doi: 10.1093/intqhc/mzm042.

| **Topic Guide** | **Item No.** | **Guide Questions/Description** | **Reported on Page No.** |
| --- | --- | --- | --- |
| **Domain 1: Research team and reflexivity** | | | |
| Personal characteristics | | | |
| 1. | Interviewer/facilitator | Which author/s conducted the interview or focus group? | 196-198 |
| 2. | Credentials | What were the researcher's credentials? e.g., PhD, MD | N/A |
| 3. | Occupation | What was their occupation at the time of the study? | N/A |
| 4. | Gender | Was the researcher male or female? | 5F/3M |
| 5. | Experience and training | What experience or training did the researcher have? | N/A |
| Relationship with participants | | | |
| 6. | Relationship established | Was a relationship established prior to study commencement? | 173-180 |
| 7. | Participant knowledge of the interviewer | What did the participants know about the researcher? e.g., personal goals, reasons for doing the research | N/A |
| 8. | Interviewer characteristics | What characteristics were reported about the interviewer/facilitator? e.g., Bias, assumptions, reasons and interests in the research topic | N/A |
| **Domain 2: study design** | | | |
| Theoretical framework | | | |
| 9. | Methodological orientation and Theory | What methodological orientation was stated to underpin the study? e.g., grounded theory, discourse analysis, ethnography, phenomenology, content analysis | 183 |
| Participant selection | | | |
| 10. | Sampling | How were participants selected? e.g., purposive, convenience, consecutive, snowball | 176 |
| 11. | Method of approach | How were participants approached? e.g., face-to-face, telephone, mail, email | 177 |
| 12. | Sample size | How many participants were in the study? | 36, 260 |
| 13. | Non-participation | How many people refused to participate or dropped out? Reasons? | N/A |
| Setting | | | |
| 14. | Setting of data collection | Where was the data collected? e.g., home, clinic, workplace | 191-192 |
| 15. | Presence of non-participants | Was anyone else present besides the participants and researchers? | 195-198 |
| 16. | Description of sample | What are the important characteristics of the sample? e.g., demographic data, date | 260-267 |
| Data collection | | | |
| 17. | Interview guide | Were questions, prompts, guides provided by the authors? Was it pilot tested? | 198-206 |
| 18. | Repeat interviews | Were repeat interviews carried out? If yes, how many? | N/A |
| 19. | Audio/visual recording | Did the research use audio or visual recording to collect the data? | 229 |
| 20. | Field notes | Were field notes made during and/or after the interview or focus group? | 209 |
| 21. | Duration | What was the duration of the interviews or focus group? | 213 |
| 22. | Data saturation | Was data saturation discussed? | 494-496 |
| 23. | Transcripts returned | Were transcripts returned to participants for comment and/or correction? | No |
| **Domain 3: analysis and findings** | | | |
| Data analysis | | | |
| 24. | Number of data coders | How many data coders coded the data? | 236-237 |
| 25. | Description of the coding tree | Did authors provide a description of the coding tree? | N/A |
| 26. | Derivation of themes | Were themes identified in advance or derived from the data? | 234 |
| 27. | Software | What software, if applicable, was used to manage the data? | N/A |
| 28. | Participant checking | Did participants provide feedback on the findings? | N/A |
| Reporting | | | |
| 29. | Quotations presented | Were participant quotations presented to illustrate the themes / findings? Was each quotation identified? e.g., participant number | 251-367 |
| 30. | Data and findings consistent | Was there consistency between the data presented and the findings? | N/A |
| 31. | Clarity of major themes | Were major themes clearly presented in the findings? | 269-272 |
| 32. | Clarity of minor themes | Is there a description of diverse cases or discussion of minor themes? | N/A |
