# Supplementary material for: Tuberculosis related barriers and facilitators among immigrants in Atlantic Canada: A qualitative study
Source: PLOS Glob Public Health. 2023 Jun 5;3(6):e0001997. doi: 10.1371/journal.pgph.0001997 (PMC10241363; doi:10.1371/journal.pgph.0001997)
Supplement: Attachment — Submitted filename: Response to Reviewers.docx [file pgph.0001997.s003.docx]

Responses to PLOS Global Public Health Reviewer’s comments

Reviewer #2: The authors did significant work to improve the quality and clarity of the manuscript since my first review. However, the manuscript still requires extensive edits.

| Reviewer #2: Comments | Authors’ Response |
| --- | --- |
| When I went to check the data availability at the University of New Brunswick, I was unable to find the data when I search for both the manuscript title or the name of the first author. The DOI link is invalid. | We are unsure why this did not work. To avoid a similar occurrence, the data supporting the information in this manuscript is now added to this manuscript as supplementary 1 (S1). |
| Line 94- missing a comma in 1,303. | This has been added (see line 94). |
| Lines 174-180- The number of participants recruited through each strategy should be made clear. How this study implemented the strategy of sampling adequacy is not described sufficiently in the methods section. | This has been noted on Line 177-180. A purposive sampling was done for the focus groups (n=14) based on their behaviours during the individual interviews (n=43) (see reference 71). Some individuals had all three criteria listed on Lines 180-183; some only met one criterion, therefore we just report the total sample size of 14 to avoid confusion when those numbers for each criteria would not add up. |
| Table 1- You have described region of origin, not countries so the label on page 12 needs to be updated. Secondly, it is unclear for individuals who are not familiar with the Canadian healthcare system whether these individuals have been linked to, assigned to, or selected a primary healthcare practitioner or whether they are in fact working as healthcare providers themselves. | Noted – changed to “Region of Origin” as advised and “Has an assigned primary healthcare provider” (see page 13). |
| Line 271-272- This sentence should be in the methods and not the results. | This sentence was rewritten to clarify the themes were the result of analyzing the data, hence the result of employing the method of inductive thematic analysis  “Four major themes emerged from the focus group data that was collected. The themes related to in relationship to barriers to testing and treatment emerged from the FGDs: 1) need for education…” (see lines 273-275). |
| Lines 228-240- You should mention more about the coding of the interview transcripts. How was this done. Was a coding tree created? What software was used to manage the data? This is also in the COREQ checklist and standard for reporting qualitative research. | A formal coding tree/book was not generated other than notes taken by each researcher.  Software was not used – analysis was conducted manually.  Lines 238-244 rewritten to now include more detail “Data were manually analyzed using inductive thematic analysis [79]. The method involves six stages: familiarization with the data by reviewing all transcripts, highlighting core concepts in similar colours and generating initial codes. Codes were then reviewed for similarities and groupings of highly related initial codes were merged together. Coders initially worked independently when identifying preliminary codes and conferred when reviewing early emerging themes. Initial themes were shared and discussed with a third coder, culminating with more clearly defined themes that were named and produced the final thematic report.” |
| Line 280- This provides insufficient information about the information that the participants were provided about the interviewers. This is a component of the COREQ checklist and can easily be added to the manuscript. | One of the strengths of this project was the cultural diversity of the research assistants and researchers working with the focus groups. This has been noted more clearly now on Lines 277-282  “Discussion facilitators were research assistants, both immigrants, who introduced themselves to the participants before starting the focus group, each explaining their own roles in the study. The lead researcher, also a foreign born Canadian, was also present in the event a question was raised that the research assistants would not feel capable of answering. They also assisted in clarification of questions when audio equipment malfunctioned for the lead interviewer in one focus group discussion. A translator was available if needed.” |
| Table 2- The table lists seven themes that are not discussed as themes in the manuscript. This inconsistency should be clarified. This table also does not represent the entire sample well. Only four individuals asked questions and 6/10 questions come from one individual. | This was spoken about in the text as gaps in the knowledge, e.g., Line 318-332, 418, 430, 487. We have edited the column header (Knowledge Gaps) to reflect how this is discussed in the text.  Correct, this table is not representative of the entire sample as we only reported on those who asked questions during the focus group. All participants were given the opportunity to ask questions throughout the discussion but only these individuals raised questions. |
| Line 364- I appreciate that the authors have worked to clarify whether an individual was discussing active TB or LTBI throughout the manuscript. However, in this line, it seems odd to mention LTBI and hospitalization. In which context would people be hospitalized for LTBI? | This sentence was referring to the treatment literature more broadly that has established more simple/less invasive/less expensive treatments have higher likelihood of engagement and behavioural adherence. However, it was not referenced and hence only added confusion in the discussion of LTBI and active TB. which you aptly pointed out. The sentence has been removed. We added clarification in Line 389 “…information available to them, e.g., LTBI and active TB are treatable, immigrants...” |
| Lines 394-424- You are presenting new data in the discussion. All quotations should be first presented in the results section. | Correct – thank you. All quotes referred to in the Discussion have now been included in the Results section.  The following was added “Participants suggested increasing engagement with available treatment stating “First awareness [of LTBI and free health care], and if I get tested because it’s free, and I have it [LTBI], then I get treated, because the treatment is available, [then] maybe [I’ll consider] the doctors, maybe the hospital” (P.4.2) (Lines 374-377).  Added to Lines 318-332 “In order to address knowledge gaps, participants suggested being proactive in disseminating accurate TB information “…through what the [YMCA] Immigrants Centre does for the immigrants [with other information], whatever way to do it [it needs to be done] more proactively. “[Don’t wait for] …just when it is detected.” P.1.3. An example of primary caregivers stepping into the role of educators was offered by another participant, “When someone is diagnosed with latent TB, to educate the person a little bit more [than they are already doing. That is not enough]. The doctor did tell me, but more information would have helped in terms of keeping you calm…I ended up doing a lot of Google search when I was in the hospital” (P.3.1). Participants volunteered to be part of future community initiatives in local settings stating “having different people from different countries, cultural beliefs, and languages [relay this information to the immigrants].” as well as saying “I want to go back to my country and gather the people and educate them” (P.5.1) and “I would volunteer [to educate them] and I know it would do a lot for the community.” (P.3.1). They also raised literacy issues when considering educational outreach programs stating “The government can put as [many] posters out there, but if you can’t read and you don’t understand, what do you do?” (P.1.2). |
| This manuscript would still benefit from a reflexivity statement. In the response comments, the authors state that reflexivity has been addressed through lines 195-198. This is not a reflexivity statement. | Added in Lines 512-561.  **Authors Personal Reflexivity**  Our research team has been together for four years, with an interdisciplinary strength drawing from medicine, public health, epidemiology, nursing, psychology, diagnostics, population health, and infectious diseases ranging from seasoned researchers to undergraduate medical and nursing students. We are all drawn by our common interest in vulnerable populations including immigrants to our local community. We come with a variety of research backgrounds including quantitative, mix-methods, meta-analyses, systematic reviews, and qualitative approaches. Our diverse team has expertise in various key areas relevant to this study, including research design, community engagement, immigrant health, policy implementation, infectious diseases, health inequities, and equity, diversity, and inclusion, to name a few. Below are key details outlining the capacity and credentials of our interdisciplinary team, and our ability to successfully implement our study: Dr Isdore Chola Shamputa holds a Ph.D. in Medical Sciences from the Vrije Universiteit Brussel in Belgium and has many years of mycobacteriology research experience across Africa, Europe, Asia, and North America. He has advanced training to postdoctoral level in TB research from the National Institutes of Health in the United States. Dr. Shamputa is also a registered nurse who has experience caring for patients from various backgrounds, including immigrants. As an Associate Professor in the Department of Nursing & Health Sciences at the University of New Brunswick, he has dedicated time for research and mentoring undergraduate and graduate students. Dr. Shamputa brings a wealth of experience leading various qualitative and quantitative research projects and has expertise in public health, epidemiology, project management, research design, and data analysis Dr Moira Law holds a PhD in Psychology trained at Carleton University, Ottawa, Ontario, Canada and subsequently working in and with a variety of departments of the federal government. She has conducted several meta-analyses and is intensely interested in identifying best practices through the systematic synthesis of the peer reviewed published literature. She deeply values the voices that speak through qualitative researchers’ work. She is motivated to make visible marginalized persons in our prisons, institutions, and communities. She has conducted several meta-analyses and is intensely interested in identifying best practices through the systematic synthesis of the peer reviewed published literature. She deeply values the voices that speak through qualitative researchers’ work. She is motivated to make visible marginalized persons in our prisons, institutions, and communities. Dr. Duncan Webster is a Royal College of Canada certified medical microbiologist, and the medical director of the level 3 laboratory at the Saint John Regional Hospital in New Brunswick. He also works in the Division of Infectious Diseases, Department of Medicine, Saint John Regional Hospital where he provides clinical care for infectious diseases, including treating LTBI and active TB patients. In addition, he is an Associate Professor at Dalhousie Medicine New Brunswick. Clara Kelly is a member of the 2023 graduating class of UNB’s Bachelor of Nursing program. As an undergraduate student, she worked on several qualitative research projects with senior members of our team. Dr. Duyen Nguyen holds a Ph.D. in population and public health and completed a 2-year Canadian Institute of Health Research Health Systems Impact Post-Doctoral Fellowship. Dr. Nguyen brings a wealth of experience leading various qualitative and quantitative research projects, and has expertise in public health, social determinants of health, project management, research design, data analysis, scaling-up health interventions, and equity, diversity, and inclusion. Dr. Tatum Burdo is a graduate from Dalhousie Medicine New Brunswick, and also holds a Master in Public Health. Dr. Jabran Umar is also a graduate from Dalhousie Medicine New Brunswick. Both Tatum and Jabran are currently completing their residency training. Dr. Kimberly Barker is a member of the Royal College Physicians and Surgeons of Canada in Public Health and Preventative Medicine, and an Associate Professor at Dalhousie Medicine New Brunswick supervising several medical students who have an interest in TB in immigrant populations. She also holds a Master in Public Health and is currently the Region Medical Officer of Health responsible for southern New Brunswick. She brings many years of experience as a physician, with Public Health training and has managed TB programs in Canada and internationally. Our team hold varied experience working in clinical settings, government settings, and academia with different goals on different projects. We recognize our collective expertise and subjectivities come with biases that we hope can still serve well those who are being studied in this project. |
| COREQ checklist- The authors have insufficiently used these reporting guidelines. Q2, Q3, Q5. Researchers credentials, education and experience could be included in the manuscript. | Done – added in Personal Reflexivity Statement |
| Q7. I addressed this in the comment above. | Noted |
| Q18 & Q23.- Why did you sometimes answer N/A vs. No? If you did not include repeat interviews then this can be easily addressed both in the manuscript and the checklist. | Adressed in lines 215-216 and 236-237. |
| Q30. I would say that you did in fact do this. When you illustrate the discussion with the data presented in the results, it can be listed here. | Noted. |
| Q32. I would also say that you discuss minor themes in your results section. I do not see how the response of not applicable applies to the discussion of minor themes. | As noted, these were not minor themes but identified and addressed as specific knowledge gaps under the need for education theme. |

**COREQ (COnsolidated criteria for REporting Qualitative research) Checklist**

Adapted from: Tong A, Sainsbury P, Craig J. Consolidated criteria for reporting qualitative research (COREQ): a 32-item checklist for interviews and focus groups. Int J Qual Health Care. 2007;19(6):349-57. doi: 10.1093/intqhc/mzm042.

| **Topic Guide** | **Item No.** | **Guide Questions/Description** | **Reported on Page No.** |
| --- | --- | --- | --- |
| **Domain 1: Research team and reflexivity** | | | |
| Personal characteristics | | | |
| 1. | Interviewer/facilitator | Which author/s conducted the interview or focus group? | 197-198 |
| 2. | Credentials | What were the researcher's credentials? e.g., PhD, MD | 522-558 |
| 3. | Occupation | What was their occupation at the time of the study? | 522-558 |
| 4. | Gender | Was the researcher male or female? | 5F/3M |
| 5. | Experience and training | What experience or training did the researcher have? | 522-558 |
| Relationship with participants | | | |
| 6. | Relationship established | Was a relationship established prior to study commencement? | 174-182 |
| 7. | Participant knowledge of the interviewer | What did the participants know about the researcher? e.g., personal goals, reasons for doing the research | 277-282 |
| 8. | Interviewer characteristics | What characteristics were reported about the interviewer/facilitator? e.g., Bias, assumptions, reasons and interests in the research topic | 511-561 |
| **Domain 2: study design** | | | |
| Theoretical framework | | | |
| 9. | Methodological orientation and Theory | What methodological orientation was stated to underpin the study? e.g., grounded theory, discourse analysis, ethnography, phenomenology, content analysis | 37, 238 |
| Participant selection | | | |
| 10. | Sampling | How were participants selected? e.g., purposive, convenience, consecutive, snowball | 177 |
| 11. | Method of approach | How were participants approached? e.g., face-to-face, telephone, mail, email | 178 |
| 12. | Sample size | How many participants were in the study? | 36, 264 |
| 13. | Non-participation | How many people refused to participate or dropped out? Reasons? | None |
| Setting | | | |
| 14. | Setting of data collection | Where was the data collected? e.g., home, clinic, workplace | 193-194 |
| 15. | Presence of non-participants | Was anyone else present besides the participants and researchers? | 197-199 |
| 16. | Description of sample | What are the important characteristics of the sample? e.g., demographic data, date | 264-271 |
| Data collection | | | |
| 17. | Interview guide | Were questions, prompts, guides provided by the authors? Was it pilot tested? | 200-212 |
| 18. | Repeat interviews | Were repeat interviews carried out? If yes, how many? | No (215-216) |
| 19. | Audio/visual recording | Did the research use audio or visual recording to collect the data? | 230 |
| 20. | Field notes | Were field notes made during and/or after the interview or focus group? | 211 |
| 21. | Duration | What was the duration of the interviews or focus group? | 215 |
| 22. | Data saturation | Was data saturation discussed? | 571-574 |
| 23. | Transcripts returned | Were transcripts returned to participants for comment and/or correction? | No (236-237) |
| **Domain 3: analysis and findings** | | | |
| Data analysis | | | |
| 24. | Number of data coders | How many data coders coded the data? | 234 |
| 25. | Description of the coding tree | Did authors provide a description of the coding tree? | No (no formal coding tree was developed) |
| 26. | Derivation of themes | Were themes identified in advance or derived from the data? | 37, 238 |
| 27. | Software | What software, if applicable, was used to manage the data? | 238 |
| 28. | Participant checking | Did participants provide feedback on the findings? | No (236-237) |
| Reporting | | | |
| 29. | Quotations presented | Were participant quotations presented to illustrate the themes / findings? Was each quotation identified? e.g., participant number | 253-387  436-451 |
| 30. | Data and findings consistent | Was there consistency between the data presented and the findings? | Yes: 324-499  526-584 |
| 31. | Clarity of major themes | Were major themes clearly presented in the findings? | 273-275 |
| 32. | Clarity of minor themes | Is there a description of diverse cases or discussion of minor themes? | No (we did not have minor themes) |
